# Educational booklet on labor and delivery: validity study

**DOI:** 10.1590/0034-7167-2024-0138

**Published:** 2024-12-13

**Authors:** Michelle Tatiane Carvalho Gonçalves, Ana Roberta Vilarouca da Silva, Mara Cristina Ribeiro Furlan, Bruna Moretti Luchesi, Tatiana Carvalho Reis Martins

**Affiliations:** I Universidade Federal de Mato Grosso do Sul. Três Lagoas, Mato Grosso do Sul, Brazil; II Universidade Federal do Piauí. Picos, Piauí, Brazil; III Universidade Federal de Mato Grosso do Sul. Campo Grande, Mato Grosso do Sul, Brazil

**Keywords:** Health Education, Validation Studies, Prenatal Care, Women’s Health, Humanized Childbirth, Educación en Salud, Estudio de Validación, Atención Prenatal, Salud de la Mujer, Parto Humanizado

## Abstract

**Objectives:**

to develop and validate an educational booklet on labor and delivery for pregnant women.

**Methods:**

this methodological study involved constructing and validating a booklet based on Echer’s framework. We used the Content Validity Index and Cronbach’s alpha for content and face validation, selecting judges according to Fering’s criteria. We then conducted a clinical validation with the target population.

**Results:**

the booklet, developed based on evidence from an integrative review and validated by judges and the target audience, achieved global Content Validity Index of 0.919 and 0.913, respectively. After clinical validation with 22 pregnant women, it included 28 topics and 48 pages, with illustrations by a graphic designer.

**Conclusions:**

expert judges and the target audience considered this educational technology valid, deeming it a relevant tool for promoting the health of pregnant women.

## RESUMEN

**Objetivos:**

construir y validar cartilla educativa sobre trabajo de parto y parto para embarazadas.

**Métodos:**

estudio metodológico, con construcción y validación de una cartilla basada en el referencial de Echer. La validez de contenido y apariencia adoptó el Índice de Validez de Contenido y el alfa de Cronbach, habiendo jueces seleccionados por el criterio de Fering. Déspues, realizada la validación clínica con la población objetivo.

**Resultados:**

la cartilla, construida con base en evidencias de una revisión integrativa, validada con jueces y público objetivo, obtuvo Índice de Validez de Contenido global de 0,919 y 0,913 respectivamente. Después de validación clínica con 22 embarazadas, fue presentada en 28 tópicos y 48 páginas, con ilustraciones producidas por designer gráfico.

**Conclusiones:**

la presente tecnología educativa fue considerada válida por los jueces especialistas y por el público objetivo, por lo tanto es considerada una herramienta relevante en la promoción de la salud de las embarazadas.

## INTRODUCTION

Pregnancy is characterized as a period of significant physical and psychological changes in a woman’s life, resulting in increased vulnerability and emotional sensitivity^(1)^. During prenatal care, women and their families can address their concerns, minimize anxieties and create a safer path throughout pregnancy^(2)^. Adequate care during pregnancy is essential for a positive outcome and the maintenance of maternal and fetal well-being, promoting health and preventing complications^(3)^.

In Brazil, healthcare professionals, primarily physicians, provide most prenatal care. This model results in highly medicalized and interventionist care. Current research indicates low adherence to these professionals’ best practices in labor and delivery care^(4,5)^. As a consequence, prenatal consultations, which should be a time for perinatal education, are not being utilized to their full potential. Several weaknesses have been identified in these consultations, such as a lack of rapport between pregnant women and healthcare providers, quick and generic consultations due to high demand in health units, and the absence of other professional categories trained to attend to this specific population^(6)^.

During prenatal care, pregnant women undergo unnecessary monthly examinations and hyper medicalization. Hospital routines prevent women from eating, walking, being in the presence of their companion, and accessing non-pharmacological methods of pain relief and analgesia. Furthermore, women are exposed to high rates of obstetric interventions, such as intravenous access, oxytocin, amniotomy, lithotomy, episiotomy, and cesarean sections without accurate indications. Some studies suggest dissatisfaction among women regarding the care received during labor and delivery-reports of abuse, disrespect, and mistreatment are common in health services^(7,8)^.

Considering the need to provide qualified and humanized care, it is essential to establish actions that help women and their families fully and positively experience pregnancy, childbirth, and postpartum. Specifically, access to quality information during prenatal care can be an effective tool, contributing to positive childbirth and birth outcomes, as well as reducing feelings of fear, anxiety, and suffering^(2)^.

Establishing educational strategies in prenatal care, such as creating a booklet, can minimize understanding difficulties. This facilitates clearer communication between women and professionals, ensuring more welcoming care that meets the needs related to pregnancy and bringing confidence, security, autonomy, and protagonism to women^(2)^.

In the context of women’s health during the gestational and puerperal cycle, it is crucial to develop and validate an easy-to-understand and accessible printed educational material (booklet) with relevant themes on the stages of labor and delivery^(9)^. Validation is an important process to ensure the production of high-quality educational technologies with language accessible to the target audience and achieving the proposed objectives^(10)^.

As an information tool with clear guidelines, the booklet can support the creation and development of educational actions throughout the gestational-puerperal cycle, proving to be a powerful reinforcement tool for verbalized guidance by health professionals^(10)^. Positive results have been observed, such as increased satisfaction, knowledge, and adherence to the treatment process and self-care after using printed materials, positively impacting the professional-patient relationship^(11)^. Additionally, educational materials serve as emancipatory technologies, as they empower individuals by providing information and enhancing communication and guidance between the health team, patients, and families^(9)^.

Given the above, this study hypothesizes that this educational technology will contribute to qualified prenatal consultations, leading to positive childbirth outcomes and empowering women to take a leading role in this process. Considering the need for changes in obstetric care related to labor and delivery, we expect positive effects, as women will become agents of transformation within hospital units based on their perception and knowledge of the parturition process, encouraging healthcare professionals to seek knowledge based on best practices in childbirth care.

## OBJECTIVES

To develop and validate an educational booklet on labor and delivery for pregnant women.

## METHODS

### Ethical Aspects

This project complies with the terms of Resolution No. 466/2012 of the National Health Council. The Research Ethics Committee of the Federal University of Mato Grosso do Sul approved this research. All study participants signed the Informed Consent Form (ICF) online and in writing.

### Study Design and Period

We conducted a methodological study to validate an educational booklet. We carried out the research between September 2022 and May 2023 in four stages: 1) literature review; 2) development of illustrations, layout, design, and texts; 3) face and content validation of the educational material by expert judges; and 4) validation of the material by representatives of the target audience^(12)^. The study was structured according to the Revised Standards for Quality Improvement Reporting Excellence (SQUIRE 2.0)^(13)^.

### Development Stages

In the first stage, we conducted a literature review to support the development of the booklet, ensuring its use as an educational technology is safe and backed by evidence. We used recommendations from the WHO, the Brazilian Association of Obstetricians and Obstetric Nurses (ABENFO), the National Guidelines for Vaginal Delivery^(14)^, the National Committee for Technology Incorporation (CONITEC) of the Unified Health System (SUS), Guidelines for Care of Pregnant Women: Cesarean Section^(15)^, the American Association of Birth Centers (AABC), the Royal College of Obstetricians and Gynaecologists (RCOG), and the National Institute for Health and Clinical Excellence (NICE) to define the topics covered in the booklet.

In the **s**econd stage, we developed the content outline, which included important information about labor and delivery, along with suggestions for illustrations that effectively represent the content and target audience characteristics. We ensured the technical language was adapted to a more popular language to facilitate understanding by pregnant women. The booklet’s layout and text structuring were based on recommendations for writing and formatting educational technologies^(16)^. Illustrations were sourced from the free illustration and image bank of the CANVA application and the Instagram profile of physiotherapist Laura Delega (@assoalhopelvico) with authorization.

In the third stage, expert judges validated the face and content of the educational material. We determined the sample size for the number of judges using the formula *n = Za^2^ × P(1-P) / e^2^*. The specified values were “Za” (confidence level) = 95%, “P” (proportion of agreement among judges) = 85%, and “e” (accepted difference from what is expected) = 15%^(17)^. The final calculation was *n = (1.962 × 0.85 × 0.15) / 0.152*, resulting in 22 judges.

We selected participants involved in teaching, care, and research related to obstetric nursing based on adaptations of the Fehring model^(18)^. Selection criteria for expert judges included: specialization or residency in obstetrics (3 points), at least one year of experience in obstetric care (2 points), teaching experience in obstetrics (2 points), publication in the field of obstetrics (2 points), a master’s degree (3 points), and participation as an attendee in obstetrics training (2 points)^(18)^. We consulted the Lattes Curriculum (available online on the Lattes Platform) to verify the expert’s adherence to the study criteria. Those who scored at least seven points were selected. Initial study participants were part of a WhatsApp group of obstetric nurses and gynecologists from the state of Mato Grosso do Sul. All received the invitation. Those interested in participating were contacted privately via WhatsApp or email and were asked to recommend other potential judges.

We sent the invitation letter via email (personal or institutional) or WhatsApp. Judges who accepted received (via email or WhatsApp) a version of the booklet, the validation instrument, and a copy of the Informed Consent Form (ICF). We developed the validation instrument using Google Forms.

The data collection instrument for the judges was divided into two parts. The first part contained questions related to profile characterization, including socioeconomic data and professional data related to practice, training, scientific production, and teaching in obstetrics. The second part concerned the booklet validation. The instrument was based on other studies^(2,19)^ and consisted of 52 items distributed across seven evaluative aspects: content, language, relevance, illustrations, layout, motivation, and culture. Each item was evaluated on a Likert scale with four options: 1) strongly disagree, 2) partially agree, 3) agree, and 4) strongly agree^(2,19)^. At the end of the instrument, we asked judges to provide general comments about the booklet. Data collection occurred between September and December 2022.

In the fourth stage, we conducted clinical validation with pregnant women. The number of invitees for this evaluation was determined using the same formula as the sample size for judges (n = 22). Inclusion criteria were: being over 18 years old, being a pregnant woman receiving care at the Family Health Units in Três Lagoas, Mato Grosso do Sul, and being able to read and write. We excluded pregnant women with visual or cognitive impairments that prevented them from reading the booklet. We approached participants at the units during peak times for pregnant women, who were there for medical or nursing consultations. The researcher personally invited participants. Those who accepted were given an explanation of ethical concepts and the consent form on the spot. Additionally, they received a pen and the printed material in a plastic folder (ICF, validation instrument, and booklet).

Data collection with the target audience took place between April and May 2023. We provided pregnant women with a printed copy of the booklet, the questionnaire, and the ICF. The questionnaire included characterization questions and an adapted version of the Suitability Assessment of Materials (SAM). The SAM consists of 22 questions covering the following areas: 1) content, 2) literacy demand, 3) illustrations, 4) layout and presentation, 5) stimulation/motivation for learning, and 6) cultural appropriateness. Items were evaluated on a Likert scale (0 = inadequate, 1 = partially adequate, and 2 = adequate)^(20,21)^.

### Data Analysis

We downloaded the data from the expert judges into Excel and exported it to the Statistical Package for the Social Sciences (SPSS), version 26.0. We manually entered the data from the target audience into Excel and then exported it to SPSS. We performed descriptive statistics using relative frequency, mean, and standard deviation. To analyze the validation data of the educational material, we calculated the Content Validity Index (CVI) for each item on the scale; then, we calculated the average CVI among the items and the overall CVI. We considered a CVI of 80% or higher as the validity parameter^(22)^. We used Cronbach’s alpha to measure the internal consistency of the instrument. A Cronbach’s alpha coefficient between 0.6 and 0.7 indicates an acceptable level of reliability, while coefficients of 0.8 or higher indicate a very good level of reliability^(23)^.

## RESULTS

### Development of the Educational Booklet

In the first stage, we searched literature by reviewing materials developed by the Ministry of Health, WHO, national and international gynecology and obstetrics colleges, and national and international obstetric nursing associations. We began the data collection with the Ministry of Health guidelines, as this institution leads Brazil’s healthcare practice guidelines. We also searched databases, initially identifying 1,060 articles. After selection, we reviewed the abstracts, resulting in a final sample of 32 articles. The eligibility criteria included: best practices in delivery and labor care, humanizing delivery, and best practices in newborn care. We searched through the Coordination for the Improvement of Higher Education Personnel (CAPES) journal portal, in the databases of Medical Literature Analysis and Retrieval System Online (MEDLINE), Latin American Literature in Health Sciences (LILACS), National Library of Medicine - National Institutes of Health (PubMed), Scopus, and Web of Science (WoS). We used the advanced search method, categorizing by title, abstract, and subject. After reading, we began the textual development, aiming to provide detailed content while maintaining accessible language for the target audience.

**Figure 1:**
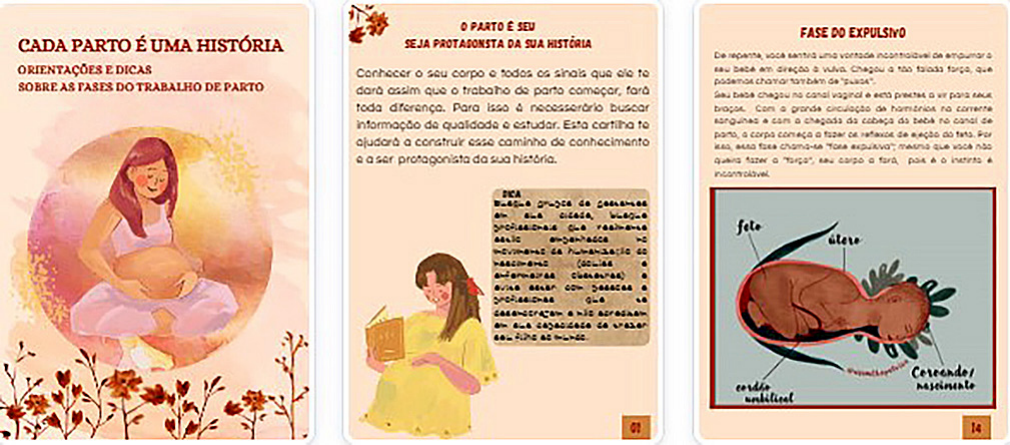
Cover and pages of the educational booklet produced and validated in this study, Três Lagoas, Mato Grosso do Sul, Brazil, 2023

In the second stage, we organized the topics and content of the educational material and chose the title. The booklet was titled “EACH BIRTH IS A STORY: Guidelines and Tips on the Stages of Labor.” It consisted of a cover and 47 pages formatted to 21 cm in height and 15 cm in width (Figure 1). We presented it in 23 topics: the birth is yours, be the protagonist of your story; the baby’s arrival; what to know about prodromal labor; mucus plug; birth plan; latent opening phase; active opening phase; curiosity: how to protect yourself from neocortical stimuli; laborland - transition phase; expulsive phase and amniotic sac; no need to cut for birth - episiotomy; what if it tears? Laceration; placental expulsion - birth of the placenta; pain in labor; trust in labor; non-pharmacological methods for pain relief; false indications for cesarean section; accurate indications for cesarean section; first contact - skin-to-skin; how the baby is right after birth; a good golden hour and the first hours after birth. To enhance interactivity, we included a birth plan template, a guide for a smoother postpartum period, a checklist for what to bring to the maternity ward, and two lists with recommendations for movies, documentaries, and YouTube channels related to childbirth and maternity.

The third stage involved selecting illustrations, adapting the vocabulary, and finishing with the layout. We chose dynamic and attractive illustrations and actual photos of births that portrayed the reality of best practices in childbirth care. We took care to select high-quality and colorful images. These were sourced from the free illustration and image bank of the CANVA app, the Instagram profile of physiotherapist Laura Della Negra (@assoalhopelvico) with permission, and the author’s collection of birth assistance, also with image use authorization. We aimed to use easily understandable words consistent with the textual message for the target audience. The booklet’s layout and textual structure followed recommendations for writing and formatting educational technologies^(16)^.

### Expert Judges Validation

In this stage, 23 expert judges participated in the evaluation of the educational material. Most of the professionals were female (90.9%), married or living with a partner (60.9%), with an average age of 37.5 years (SD = 9.19). Regarding their state of practice, most were from Mato Grosso do Sul (78.3%), followed by Paraíba (8.7%), Minas Gerais (4.3%), Rio Grande do Norte (4.3%), and São Paulo (4.3%). All had specialized in gynecology and obstetrics, with 95.7% having additional training in this field and 77.3% completing their most recent training in 2021/2022. In terms of academic qualifications, 30.4% had a master’s degree, and 8.7% had a doctoral degree.

In the validation process of the educational technology, all items met the minimum approval criteria (CVI ≥ 80%), with the CVI exceeding 0.91 in six of the seven evaluated categories. With an overall CVI of 0.91, we considered the educational material validated (Table 1).

**Table 1:** Evaluation of the Content Validity Index and assessment of internal consistency of nurses’ responses (N = 23), Três Lagoas, Mato Grosso do Sul, Brazil, 2023

	SD	PD	A	SA	CVI	Cronbach’s Alfa
	n	n	n	n		
1. Scientific Accuracy					0.942	0.879
1.1 Do the contents align with current knowledge?	0	1	6	16	0.957	0.846
1.2 Are the presented guidelines necessary and correctly addressed?	0	1	11	11	0.957	0.872
1.3 Are technical terms adequately defined?	0	2	6	15	0.913	0.752
2. Content					0.930	0.857
2.1 Are the information objectives evident (to provide clear information to pregnant women)?	0	2	5	16	0.913	0.822
2.2 Are the information satisfactory regarding the desired behavior?	0	1	10	12	0.957	0.814
2.3 There is no unnecessary information.	1	2	11	9	0.870	0.785
2.4 Key points are reviewed.	0	2	9	12	0.913	0.835
2.5 Infractions are updated.	0	0	5	18	1.000	0.903
3. Literary Presentation					0.928	0.912
3.1 Is the language neutral (without comparative adjectives, not promotional, and without false appeals)?	0	1	12	10	0.957	0.906
3.2 Is the language explanatory?	0	1	7	15	0.957	0.901
3.3 Does the material promote and encourage adherence to vaginal delivery?	0	0	7	16	1.000	0.918
3.4 Is the vocabulary mainly composed of common words?	0	2	10	11	0.913	0.907
3.5 Is the vocabulary composed of simple words?	0	2	13	8	0.913	0.897
3.6 Is the language appropriate for the target audience?	0	4	10	9	0.826	0.900
3.7 Are the ideas expressed concisely?	1	1	12	9	0.913	0.902
3.8 Does the text allow for interaction with guidance between professionals and the target audience?	0	0	9	14	1.000	0.905
3.9 Does the text allow for interaction with the logical sequence of the childbirth process?	0	0	10	13	1.000	0.915
3.10 Is the planning and sequencing of information consistent, facilitating understanding by the target audience?	0	1	10	12	0.957	0.892
3.11 Is the material pleasant to read?	0	1	11	11	0.957	0.896
3.12 Is the material of adequate length, neither extensive nor tiring?	1	5	11	6	0.739	0.910
4. Illustrations					0.930	0.889
4.1 Are the illustrations simple, appropriate, and easily understandable?	0	2	7	14	0.913	0.882
4.2 Are the illustrations familiar to the readers?	0	2	7	14	0.913	0.843
4.3 Are they related to the text (fulfilling the intended purpose)?	0	1	10	12	0.957	0.850
4.5 Are they integrated into the text (well located)?	0	0	8	15	1.000	0.888
4.6 Are they self-explanatory?	0	3	8	12	0.870	0.849
5. Material Sufficiently Specific and Comprehensive					0.913	0.733
5.1 Does the material promote understanding to avoid unnecessary cesarean surgery?	0	2	6	15	0.913	0.680
5.2 Does it provide maximum information to reduce unnecessary cesareans and increase vaginal delivery rates?	0	2	9	12	0.913	0.618
5.3 Are the instructions for obtaining a respectful vaginal delivery clear and understandable?	0	3	10	10	0.870	0.628
5.4 Is there no ambiguous wording in the text?	0	2	12	9	0.913	0.806
5.5 Is the content written in a style that centers on the pregnant woman?	0	1	10	12	0.957	0.678
6. Readability and Print Characteristics					0.875	0.952
6.1 Is the font size appropriate?	1	4	9	9	0.783	0.949
6.2 Is the font style appropriate?	0	2	10	11	0.913	0.946
6.3 Is the letter spacing appropriate?	0	3	8	12	0.870	0.938
6.4 Is the line length appropriate?	0	2	9	12	0.913	0.941
6.5 Is the line spacing appropriate?	0	4	9	10	0.826	0.938
6.6 Is there adequate use of white space to reduce the appearance of crowded text?	0	3	13	7	0.870	0.938
6.7 Is paragraph spacing appropriate?	0	4	12	7	0.826	0.942
6.8 Is the material format appropriate?	0	0	14	9	1.000	0.954
7. Information Quality					0.913	0.846
7.1 Is it embedded in the local culture?	0	3	14	6	0.870	0.769
7.2 Is it embedded in the current culture?	0	2	13	8	0.913	0.808
7.3 Does the material enable the target audience to perform the desired actions?	0	0	13	10	1.000	0.858
7.4 Does the material help prevent possible childbirth problems?	0	4	12	7	0.826	0.804
7.5 Does the material allow for the maximum possible benefit?	0	1	11	11	0.957	0.834
Global CVI					0.919	

*SD - strongly disagree; PD - partially disagree; A - agree; SA - strongly agree; CVI - Content Validity Index.*

Regarding internal consistency, the educational material showed very good reliability levels in six evaluated categories. Only Category 5, “Material sufficiently specific and comprehensive,” showed a Cronbach’s alpha coefficient of 0.73, representing an acceptable reliability level (Table 1).

The judges provided 27 suggestions, of which 21 were accepted: 15 related to content to improve text clarity; one related to language, suggesting the replacement of technical terms with more popular ones; four related to illustrations, suggesting the inclusion and change of illustrations to match the text better; and one related to layout, suggesting the reduction of text length and increased spacing between paragraphs for better visual cleanliness.

The judges’ suggestions and comments were invaluable, enriching and enhancing the educational material to achieve the proposed objective. They highlighted key points: the relevance of the topic, the use of recent scientific evidence, the richness of details in the information, and the easy to-understand language.

In evaluating internal consistency, even though the item “Material sufficiently specific and comprehensive” in Category 5 had a Cronbach’s alpha coefficient of 0.73 (an acceptable reliability level), the judges suggested improvements to the educational material, which we accepted.

Concerning the content of the booklet, the judges made several relevant suggestions. They recommended including the support and presence of a companion throughout the informational process during pregnancy; replacing the phrase “no professional should put their hands on the vulva” [during childbirth], as the correct term, according to the Ministry of Health’s Childbirth Assistance Guidelines, is “hands-on,” allowing the professional to choose whether or not to support the baby’s head; replacing the information about degrees of laceration with a more straightforward and easier to understand explanation; regarding placenta management, the national childbirth assistance guideline advises both active and physiological management, recommending active management to prevent postpartum hemorrhage, thus suggesting replacing “ideal” with “physiological management”; including the topic “membrane rupture (water breaking)”; and adding information about the postpartum period.

As for language, the judges suggested replacing some technical terms with simpler terms for easier understanding by the target audience: for example, replacing “assessment of fetal and maternal vitality” and “rotation of the fetus before the final delivery,” respectively, with “assessing the baby’s heartbeat and mother’s well-being” and “the baby may not come out quickly and all at once, with the head coming out first and the body in the next contraction”; replacing the word “no” in some phrases, considering the brain’s difficulty in processing this word; and conducting a textual review.

Regarding illustrations, the judges commented on the need to add image references. In terms of cultural adequacy, only one judge commented that the booklet’s proposal is not aligned with the local and current culture, as Mato Grosso do Sul and Brazil have high cesarean rates, establishing a “cesarean culture.” It is important to note that the comments and/or suggestions included pointing out spelling and agreement issues; thus, a qualified professional conducted a textual review of the material.

### Clinical Validation

We conducted this validation stage with 22 pregnant women attended by five eSFs in Três Lagoas. Most participants were married (77.3%), aged between 25 and 30, and had attended higher education (53%). Regarding parity, 14 (63.6%) were primiparas, six (27.3%) were secundiparas, and two (9.1%) were multiparas. In terms of gestational age, ten (45.5%) were in the second trimester, and 12 (54.5%) were in the third. As for obstetric data, a minority of the pregnant women (43.0%) had attended seven or more prenatal consultations, justified by the gestational age of the women undergoing prenatal care.

Of the six categories evaluated by the pregnant women, five achieved a CVI greater than 0.91, with only Category 3, “Illustrations,” scoring lower (0.84). The booklet’s total CVI was 0.91, reaching the necessary value for validation (Table 2).

**Table 2:** Content Validity Index Assessment by Pregnant Women (N = 22), Três Lagoas, Mato Grosso do Sul, Brazil, 2023

	Inadequate	Partially adequate	Adequate	CVI
	n	n	n	
1 - Content				0.932
1.a The cover caught your attention.	0	3	19	0.864
1.b The content sequence is appropriate.	0	1	21	0.955
1.c The structure of the educational booklet is appropriate.	0	1	21	0.955
1.d The content highlights the main points.	0	1	21	0.955
2 - Writing Style				0.918
2.a The sentences are easy to understand.	0	0	22	1.000
2.b The written content is clear.	0	3	19	0.864
2.c Uses common vocabulary in the text.	0	2	20	0.909
2.d Topics facilitated learning.	0	2	20	0.909
2.e The text is engaging.	0	2	20	0.909
3 - Illustrations				0.841
3.a The purpose of the illustration related to the text is clear.	0	3	19	0.864
3.b The illustrations are simple.	0	5	17	0.773
3.c The illustrations serve to complement the text.	0	2	20	0.909
3.d The figures/illustrations are relevant.	0	4	18	0.818
4 - Layout and Presentation				0.920
4.a Layout characteristics	0	1	21	0.955
4.b Font size and type	0	3	19	0.864
4.c Subtitles are used.	0	1	21	0.955
4.d The pages or sections appear organized.	0	2	20	0.909
5 - Stimulation/Motivation for Learning				0.924
5.a In your opinion, will any pregnant woman who reads this booklet understand it?	0	3	19	0.864
5.b The educational material covers the necessary topics for pregnant women to feel prepared for labor and delivery.	0	1	21	0.955
5.c You felt motivated to read the booklet to the end.	0	1	21	0.955
6 - Cultural Adequacy				0.977
6.a The material is culturally appropriate to your logic, language, and experience.	0	1	21	0.955
6.b It presents culturally appropriate images and examples.	0	0	22	1.000
Global CVI				0.913

*CVI - Content Validity Index.*

In the evaluated items, we observed that all pregnant women understood the booklet’s theme and main objective well. Almost all (95.5%) classified the booklet as necessary to feel prepared for labor and delivery. Regarding the text, all understood the phrases contained in the booklet. Additionally, most pregnant women (95.5%) showed interest in reading the booklet to the end. Furthermore, 95.5% stated that the content highlights the main points. Only one pregnant woman provided a suggestion/comment: “*The booklet dispelled many myths I had. I will definitely be more informed and calm for my second delivery*.”

After clinical validation, the final version of the booklet consisted of 48 pages, 44 of which were dedicated to content and four post-text pages.

## DISCUSSION

In this study, we produced and validated educational material as a booklet. Using validated technologies increases reliability, facilitating communication in the health teaching learning process^(24)^. Educational materials are commonly used in health care, covering various topics^(25)^. These technologies appear in various formats, such as manuals for safe surgery, educational games on adolescent depression, series albums on congenital syphilis, educational booklets for home care of premature newborns, educational leaflets for tuberculosis sputum collection, and educational videos on newborn care^(2,26)^.

These health education technologies play a significant role in health promotion. They can change individual and collective realities and lead to more informed decision-making, thus reducing potential health risks and promoting favorable outcomes^(27)^.

Conducting a literature review was essential in developing this educational material. We reviewed the latest evidence on the subject, guided by the Ministry of Health’s guidelines on childbirth assistance in Brazil. The ideas presented aligned with the target audience’s needs, achieving the proposed objective^(27)^.

The booklet proved reliable during expert validation, showing good CVI and being suitable for the target audience. Most suggestions and recommendations were accepted to improve the language and comprehension of the information presented. The unaccepted suggestions were either already mentioned or not aligned with the booklet’s proposal. Thus, the primary modifications involved adding missing topics and replacing specific technical terms with more comprehensible ones for the target audience. The pregnant women’s feedback was very positive, highlighting that they found the booklet engaging and informative.

The booklet stands out from others due to its detailed and in-depth approach, with practical tips on “what to do and what to feel” during each phase of labor, making it unique. To capture the pregnant women’s attention, we used topics like “Curiosity,” which is also present in other educational materials^(10,28)^.

In Brazil, childbirth assistance is entirely hospital-based, following a medical-centric model. As a result, this assistance becomes highly interventionist and medicalized. Current research confirms this, showing low adherence to best practices in labor and delivery assistance by healthcare professionals^(4,5)^. In hospital routines, women are prevented from eating, walking, having their companion present, accessing non-pharmacological pain relief methods, and analgesia. They are subjected to high rates of obstetric interventions, such as intravenous access, oxytocin, amniotomy, lithotomy, episiotomy, and cesarean section without accurate indication. Some studies have suggested dissatisfaction among women with the assistance received during labor and delivery, with reports of abuse, disrespect, and mistreatment being common in health services^(7,8)^.

Therefore, the educational booklet “EACH BIRTH IS A STORY: Guidelines and Tips on the Stages of Labor” is a validated educational technology, containing appropriate language, illustrations, and layout that are simple and attractive to readers. We expect this booklet to be used during prenatal care by pregnant women, contributing to improving prenatal consultations’ quality, favoring positive childbirth outcomes, and empowering women to become protagonists in this process.

This educational material also serves as an emancipatory technology, providing information that contributes to individuals’ empowerment and enhances communication and guidance processes between the healthcare team, patients, and families^(9)^.

### Study limitations

The study was limited by the difficulty of obtaining a representative sample of judges from all Brazilian states, with a higher number of participants from the Central-West Region.

### Contributions to Health

Nurses can use this educational booklet during prenatal consultations to inform pregnant women about the topics covered. Additionally, the booklet can help identify risks and vulnerabilities within this population, supporting the provision of care with education and health promotion as fundamental pillars.

## CONCLUSIONS

Expert judges and the target audience found the educational technology’s content and face relevant, with a total CVI above 0.90, indicating satisfactory values. The applied methodology effectively aided in developing the educational booklet in a didactic, dynamic, comprehensible, attractive, and dialogical manner, contributing to the knowledge of pregnant women and potentially the professionals who care for them.

The validation results by expert judges and the target audience highlight the content’s suitability to the local reality, suggesting that the material can help fill gaps in care for pregnant women. This educational material has the potential to reduce poor practices in childbirth care and decrease obstetric violence.

## Supplementary Material

0034-7167-reben-77-05-e20240138-suppl01

0034-7167-reben-77-05-e20240138-suppl02

0034-7167-reben-77-05-e20240138-suppl03

## Data Availability

https://doi.org/10.48331/scielodata.HSJMFS
