# Supplementary material for: Educational booklet on labor and delivery: validity study
Source: Rev Bras Enferm. 2024 Dec 13;77(5):e20240138. doi: 10.1590/0034-7167-2024-0138 (PMC11654228; doi:10.1590/0034-7167-2024-0138)
Supplement: 0034-7167-reben-77-05-e20240138-suppl01 [file 0034-7167-reben-77-05-e20240138-suppl01.pdf]

Nome	IDADADE GESTACIONAL (trimestre)	Sexo	Estado Civil	PARIDADE	1.a A capa chamou sua atenção	1.b A sequência do conteúdo está adequada	1.c A estrutura da cartilha educativa está adequada	1.d O conteúdo destaca os pontos principais	2.a As frases são fáceis de entender	2.b O conteúdo escrito é claro
G01	3	Feminino	Casada	PRIMIGESTA	Adequado	Adequado	Adequado	Adequado	Adequado	Adequado
G02	2	Feminino	Solteira	PRIMIGESTA	Adequado	Parcialmente Adequado	Adequado	Adequado	Adequado	Adequado
G03	3	Feminino	Casada	SECUNDIGESTA	Adequado	Adequado	Parcialmente Adequado	Adequado	Adequado	Adequado
G04	3	Feminino	Casada	MULTIGESTA	Adequado	Adequado	Adequado	Adequado	Adequado	Parcialmente Adequado
G05	3	Feminino	Casada	PRIMIGESTA	Adequado	Adequado	Adequado	Adequado	Adequado	Adequado
G06	3	Feminino	Casada	SECUNDIGESTA	Adequado	Adequado	Adequado	Adequado	Adequado	Adequado
G07	3	Feminino	Casada	PRIMIGESTA	Parcialmente Adequado	Adequado	Adequado	Adequado	Adequado	Adequado
G08	3	Feminino	Casada	PRIMIGESTA	Adequado	Adequado	Adequado	Adequado	Adequado	Adequado
G09	2	Feminino	Solteira	PRIMIGESTA	Adequado	Adequado	Adequado	Adequado	Adequado	Adequado
G10	3	Feminino	Solteira	PRIMIGESTA	Adequado	Adequado	Adequado	Adequado	Adequado	Adequado
G11	3	Feminino	Casada	PRIMIGESTA	Adequado	Adequado	Adequado	Adequado	Adequado	Adequado
G12	2	Feminino	Casada	MULTIGESTA	Adequado	Adequado	Adequado	Adequado	Adequado	Parcialmente Adequado
G13	3	Feminino	Casada	PRIMIGESTA	Adequado	Adequado	Adequado	Parcialmente Adequado	Adequado	Adequado
G14	2	Feminino	Casada	PRIMIGESTA	Adequado	Adequado	Adequado	Adequado	Adequado	Adequado
G15	2	Feminino	Casada	SECUNDIGESTA	Adequado	Adequado	Adequado	Adequado	Adequado	Adequado
G16	2	Feminino	Casada	SECUNDIGESTA	Adequado	Adequado	Adequado	Adequado	Adequado	Adequado
G17	2	Feminino	Casada	PRIMIGESTA	Parcialmente Adequado	Adequado	Adequado	Adequado	Adequado	Adequado
G18	2	Feminino	Casada	PRIMIGESTA	Parcialmente Adequado	Adequado	Adequado	Adequado	Adequado	Adequado
G19	2	Feminino	Casada	PRIMIGESTA	Adequado	Adequado	Adequado	Adequado	Adequado	Parcialmente Adequado
G20	3	Feminino	Casada	SECUNDIGESTA	Adequado	Adequado	Adequado	Adequado	Adequado	Adequado
G21	3	Feminino	Solteira	PRIMIGESTA	Adequado	Adequado	Adequado	Adequado	Adequado	Adequado
G22	2	Feminino	Solteira	SECUNDIGESTA	Adequado	Adequado	Adequado	Adequado	Adequado	Adequado

2.c Usa vocabulário com palavras comuns no texto	2.d O aprendizado é facilitado por tópicos	2.e O texto é interessante	3.a O propósito da ilustração referente ao texto está claro	3.b As ilustrações são simples	3.c As ilustrações servem para complementar o texto?	3.d As figuras/ilustrações são relevantes	4.a Característica do layout	4.b Tamanho e tipo de letra	4.c São utilizados subtítulos
Adequado	Adequado	Adequado	Adequado	Adequado	Adequado	Adequado	Adequado	Adequado	Adequado
Adequado	Adequado	Adequado	Adequado	Parcialmente Adequado	Adequado	Adequado	Adequado	Adequado	Adequado
Adequado	Adequado	Adequado	Adequado	Parcialmente Adequado	Adequado	Adequado	Adequado	Parcialmente Adequado	Adequado
Parcialmente Adequado	Adequado	Adequado	Adequado	Adequado	Adequado	Adequado	Adequado	Adequado	Adequado
Adequado	Adequado	Adequado	Adequado	Adequado	Adequado	Adequado	Adequado	Adequado	Adequado
Adequado	Adequado	Adequado	Adequado	Adequado	Adequado	Adequado	Adequado	Adequado	Adequado
Adequado	Parcialmente Adequado	Adequado	Adequado	Adequado	Adequado	Parcialmente Adequado	Adequado	Adequado	Adequado
Adequado	Adequado	Adequado	Adequado	Adequado	Adequado	Adequado	Adequado	Adequado	Adequado
Adequado	Adequado	Adequado	Adequado	Parcialmente Adequado	Adequado	Parcialmente Adequado	Adequado	Adequado	Adequado
Parcialmente Adequado	Adequado	Adequado	Adequado	Adequado	Adequado	Adequado	Adequado	Parcialmente Adequado	Adequado
Adequado	Adequado	Adequado	Adequado	Adequado	Adequado	Adequado	Adequado	Adequado	Adequado
Adequado	Adequado	Adequado	Parcialmente Adequado	Parcialmente Adequado	Adequado	Adequado	Adequado	Adequado	Adequado
Adequado	Adequado	Adequado	Parcialmente Adequado	Parcialmente Adequado	Adequado	Adequado	Adequado	Adequado	Adequado
Adequado	Adequado	Adequado	Adequado	Adequado	Adequado	Parcialmente Adequado	Adequado	Adequado	Adequado
Adequado	Adequado	Adequado	Adequado	Adequado	Parcialmente Adequado	Adequado	Adequado	Adequado	Parcialmente Adequado
Adequado	Adequado	Adequado	Adequado	Adequado	Adequado	Adequado	Adequado	Adequado	Adequado
Adequado	Adequado	Adequado	Adequado	Adequado	Adequado	Parcialmente Adequado	Adequado	Adequado	Adequado
Adequado	Adequado	Parcialmente Adequado	Parcialmente Adequado	Adequado	Adequado	Adequado	Adequado	Parcialmente Adequado	Adequado
Adequado	Parcialmente Adequado	Adequado	Adequado	Adequado	Adequado	Adequado	Parcialmente Adequado	Adequado	Adequado
Adequado	Adequado	Adequado	Adequado	Adequado	Adequado	Adequado	Adequado	Adequado	Adequado
Adequado	Adequado	Parcialmente Adequado	Adequado	Adequado	Parcialmente Adequado	Adequado	Adequado	Adequado	Adequado
Adequado	Adequado	Adequado	Adequado	Adequado	Adequado	Adequado	Adequado	Adequado	Adequado

4.d As páginas ou seções parecem organizadas	5.a Em sua opinião, qualquer gestante que ler essa cartilha vai entender?	5.b No material educativo são abordados os assuntos necessários para que as gestantes sintam-se preparadas para o trabalho de parto e parto.	5.c Você se sentiu motivado a ler a cartilha até o final	6.a O material é culturalmente adequado à sua lógica, linguagem e experiência	6.b Apresenta imagens e exemplos adequados culturalmente	Sugestões/Comentários
Adequado	Adequado	Adequado	Adequado	Adequado	Adequado	
Adequado	Adequado	Adequado	Parcialmente Adequado	Adequado	Adequado	
Adequado	Parcialmente Adequado	Adequado	Adequado	Adequado	Adequado	
Adequado	Adequado	Adequado	Adequado	Adequado	Adequado	
Adequado	Adequado	Adequado	Adequado	Adequado	Adequado	
Adequado	Adequado	Adequado	Adequado	Adequado	Adequado	Cartilha tirou muitos mitos que tinha na minha cabeça. Com certeza terei meu segundo parto mais informada e tranquila.
Adequado	Adequado	Adequado	Adequado	Adequado	Adequado	
Adequado	Adequado	Adequado	Adequado	Adequado	Adequado	
Parcialmente Adequado	Adequado	Adequado	Adequado	Adequado	Adequado	
Adequado	Parcialmente Adequado	Adequado	Adequado	Adequado	Adequado	
Adequado	Adequado	Adequado	Adequado	Adequado	Adequado	
Adequado	Adequado	Adequado	Adequado	Adequado	Adequado	
Adequado	Parcialmente Adequado	Parcialmente Adequado	Adequado	Adequado	Adequado	
Adequado	Adequado	Adequado	Adequado	Adequado	Adequado	
Adequado	Adequado	Adequado	Adequado	Adequado	Adequado	
Adequado	Adequado	Adequado	Adequado	Adequado	Adequado	
Adequado	Adequado	Adequado	Adequado	Adequado	Adequado	
Parcialmente Adequado	Adequado	Adequado	Adequado	Adequado	Adequado	
Adequado	Adequado	Adequado	Adequado	Adequado	Adequado	
Adequado	Adequado	Adequado	Adequado	Parcialmente Adequado	Adequado	
Adequado	Adequado	Adequado	Adequado	Adequado	Adequado	
Adequado	Adequado	Adequado	Adequado	Adequado	Adequado	
