# Supplementary material for: Educational booklet on labor and delivery: validity study
Source: Rev Bras Enferm. 2024 Dec 13;77(5):e20240138. doi: 10.1590/0034-7167-2024-0138 (PMC11654228; doi:10.1590/0034-7167-2024-0138)
Supplement: 0034-7167-reben-77-05-e20240138-suppl03 [file 0034-7167-reben-77-05-e20240138-suppl03.pdf]

Nome	Data de nascimento	Sexo	Estado Civil	Você mora em qual estado do Brasil?	Formação (Graduação):	Possui Capacitação em Ginecologia e Obstetrícia?	Se sim, qual ano que participou da última capacitação ?	Possui Especializaçã o em Ginecologia e Obstetrícia?	Se sim, em qual ano concluiu a especialização em Ginecologia e Obstetrícia (ex: 1997, 2008, etc.)?	Possui Mestrado ?
J01	7/9/1977	Feminino	Solteiro (a)	Rio grande do norte	Enfermagem	Sim	2022	Sim	2015	Não
J02	3/18/1987	Feminino	Casado (a) ou vive com companheiro (a)	Mato Grosso do Sul	Enfermagem	Sim	2019	Sim	2011	Não
J03	12/31/1992	Feminino	Casado (a) ou vive com companheiro (a)	Mato Grosso do Sul	Enfermagem	Sim	2021	Sim		Não
J04	5/10/1974	Feminino	Casado (a) ou vive com companheiro (a)	Mato grosso do Sul	Enfermagem	Sim	2021	Sim	2017	Não
J05	3/22/1966	Feminino	Casado (a) ou vive com companheiro (a)	Minas Gerais	Enfermagem	Sim	2002 e 2012	Sim	2000	Não
J06	6/6/1992	Feminino	Solteiro (a)	Mato Grosso do Sul	Enfermagem	Sim	2022	Sim	2020	Sim

J07	5/5/1984	Feminino	Casado (a) ou vive com companheiro (a)	São Paulo	Enfermagem	Não		Sim	2017	Não
J08	11/20/1994	Feminino	Solteiro (a)	Mato Grosso do Sul	Enfermagem	Sim	2022	Sim	2020	Não
J09	6/19/1961	Feminino	Solteiro (a)	Mato Grosso do Sul	Enfermeira Obstetrica	Sim	2022	Sim	2020	Não
J10	4/10/1988	Feminino	Casado (a) ou vive com companheiro (a)	Mato Grosso do Sul	Enfermagem	Sim	2022	Sim	2016	Sim
J11	10/16/2022	Feminino	Casado (a) ou vive com companheiro (a)	Mato grosso do Sul	Enfermagem	Sim	Julho	Sim	2015	Não

J12	7/18/1989	Masculino	Solteiro (a)	Paraíba	Enfermagem	Sim	2021	Sim	2018	Sim
J13	3/10/1986	Feminino	Casado (a) ou vive com companheiro (a)	Mato Grosso do Sul	Enfermagem	Sim	2015	Sim	2012	Sim
J14	1/26/1979	Masculino	Casado (a) ou vive com companheiro (a)	Mato Grosso do Sul	Enfermagem	Sim	2022	Sim	2015	Não
J15	10/28/1974	Feminino	Divorciado/ separado/ desquitado	PB	Enfermagem	Sim	2019	Sim	2001	Sim
J16	6/13/1977	Feminino	Casado (a) ou vive com companheiro (a)	Mato Grosso Do Sul	Enfermagem	Sim	2022	Sim	2014	Não
J17	12/30/1985	Feminino	Solteiro (a)	MATO GROSSO DO SUL	Enfermagem	Sim	2021	Sim	2014	Sim
J18	8/15/1991	Feminino	Casado (a) ou vive com companheiro (a)	Mato Grosso do Sul	Enfermagem	Sim	2021	Sim	2022	Não

J19	7/7/1987	Feminino	Casado (a) ou vive com companheiro (a)	Mato Grosso do Sul	Enfermagem	Sim	2022	Sim	2018	Não
J20	3/25/1989	Feminino	Casado (a) ou vive com companheiro (a)	Mato Grosso do Sul	Enfermagem	Sim	2018	Sim	2018	Não
J21	2/16/1995	Feminino	Solteiro (a)	Ms	Enfermagem	Sim	2022	Sim	2022	Não
J22	6/29/1994	Feminino	Solteiro (a)	Mato Grosso do Sul	Enfermagem	Sim	2021	Sim	2021	Não
J23	4/28/1988	Feminino	Casado (a) ou vive com companheiro (a)	Mato Grosso Do Sul	Enfermagem	Sim	2022	Sim	2012	Sim

Possui Doutorado?	1.1 Os conteúdos abordados estão de acordo com o conhecimento atual?	1.2 As orientações apresentadas são as necessárias e foram abordadas corretamente?	1.3 Os termos técnicos estão adequadamente definidos?	2.1 Os objetivos das informações são evidentes (levar informação clara às gestantes).	2.2 As informações são satisfatórias quanto ao comportamento desejado?	2.3 Não existe informações desnecessárias.	2.4 Existe revisão dos pontos mais importantes.	2.5 As inforções são atualizadas.
Não	Concordo totalmente	Concordo totalmente	Concordo totalmente	Concordo totalmente	Concordo totalmente	Concordo totalmente	Concordo totalmente	Concordo totalmente
Não	Concordo	Concordo	Concordo	Concordo	Concordo	Concordo	Concordo	Concordo
Não	Concordo totalmente	Concordo	Concordo totalmente	Concordo totalmente	Concordo	Concordo	Concordo	Concordo totalmente
Não	Concordo totalmente	Concordo	Concordo totalmente	Concordo totalmente	Concordo totalmente	Concordo	Concordo totalmente	Concordo totalmente
Não	Concordo totalmente	Concordo totalmente	Concordo totalmente	Concordo totalmente	Concordo totalmente	Concordo totalmente	Concordo totalmente	Concordo totalmente
Não	Concordo totalmente	Concordo totalmente	Concordo totalmente	Concordo totalmente	Concordo totalmente	Concordo totalmente	Concordo totalmente	Concordo totalmente

Não	Concordo totalmente	Concordo totalmente	Concordo totalmente	Concordo totalmente	Concordo totalmente	Concordo totalmente	Concordo totalmente	Concordo totalmente
Não	Concordo parcialmente	Concordo	Concordo parcialmente	Concordo	Concordo	Concordo parcialmente	Concordo	Concordo totalmente
Não	Concordo totalmente	Concordo totalmente	Concordo totalmente	Concordo totalmente	Concordo totalmente	Concordo	Concordo	Concordo
Não	Concordo	Concordo	Concordo	Concordo	Concordo	Concordo	Concordo totalmente	Concordo totalmente
Não	Concordo	Concordo	Concordo totalmente	Concordo totalmente	Concordo	Concordo	Concordo	Concordo

Sim	Concordo totalmente	Concordo totalmente	Concordo totalmente	Concordo totalmente	Concordo totalmente	Concordo totalmente	Concordo totalmente	Concordo totalmente
Não	Concordo totalmente	Concordo totalmente	Concordo totalmente	Concordo totalmente	Concordo totalmente	Concordo totalmente	Concordo totalmente	Concordo totalmente
Não	Concordo totalmente	Concordo totalmente	Concordo totalmente	Concordo totalmente	Concordo	Concordo	Concordo	Concordo totalmente
Sim	Concordo	Concordo parcialmente	Concordo parcialmente	Concordo parcialmente	Concordo parcialmente	Discordo totalmente	Concordo parcialmente	Concordo totalmente
Não	Concordo totalmente	Concordo	Concordo	Concordo	Concordo	Concordo	Concordo	Concordo totalmente
Não	Concordo totalmente	Concordo totalmente	Concordo totalmente	Concordo totalmente	Concordo totalmente	Concordo totalmente	Concordo totalmente	Concordo totalmente
Não	Concordo totalmente	Concordo	Concordo totalmente	Concordo totalmente	Concordo totalmente	Concordo totalmente	Concordo totalmente	Concordo totalmente

Não	Concordo	Concordo	Concordo	Concordo	Concordo	Concordo	Concordo	Concordo
Não	Concordo totalmente	Concordo totalmente	Concordo totalmente	Concordo totalmente	Concordo totalmente	Concordo	Concordo parcialmente	Concordo totalmente
Não	Concordo	Concordo	Concordo	Concordo parcialmente	Concordo	Concordo parcialmente	Concordo	Concordo
Não	Concordo totalmente	Concordo	Concordo	Concordo totalmente	Concordo	Concordo	Concordo totalmente	Concordo totalmente
Não	Concordo totalmente	Concordo totalmente	Concordo totalmente	Concordo totalmente	Concordo totalmente	Concordo totalmente	Concordo totalmente	Concordo totalmente

Acrescente sua sugestão quanto ao conteúdo da cartilha neste espaço (por favor especifique o capítulo):	3.1 A linguagem é neutra (sem adjetivos comparativos, sem ser promocional e sem apelos inverídicos).	3.2 A linguagem é explicativa.	3.3 O material promove e encoraja à adesão ao parto normal.	3.4 O vocabulário é composto em sua maioria por palavras comuns.	3.5 O vocabulário empregado é composto por palavras simples.
Indicação real de cesárea<br>Nó verdadeiro de cordão umbilical	Concordo totalmente	Concordo totalmente	Concordo totalmente	Concordo totalmente	Concordo totalmente
	Concordo	Concordo	Concordo	Concordo	Concordo
	Concordo	Concordo totalmente	Concordo totalmente	Concordo totalmente	Concordo totalmente
Está apta para publicação	Concordo	Concordo totalmente	Concordo totalmente	Concordo totalmente	Concordo totalmente
Em nenhum momento consegui detectar o estímulo em levar o acompanhante para os momentos de preparo com informações, assim permitindo que esteja mesmo preparado para ajuda-la. No capitulo plano de parto a frase em relação à escrita do desejo quando tem a palavra "não" na frente é importante estar isolada pois sabemos que, o cérebro tem dificuldade de processar a palavra "não". Mais a frente no capitulo da fase de transição o capitulo inteiro foi repetido não entendi se foi intencionalmente. Na pagina 16 ao invés de não recomenda pode substituir por "OMS desaconselha a episiotomia", pela mesma razão do cérebro. Interessante se estimulasse o acompanhante a treinar a respiração com a mulher para o cérebro reconhecer o estimulo através do cheiro da pessoa. Acredito que seria importante escrever a porcentagem das 41 semanas na pag 23. Quanto ao capitulo do modelo de plano de parto, pag 36, acredito na confecção do plano de parto como carta de apresentação a equipe de saúde, sendo o modelo de múltipla escolha mais impessoal e por isso menos envolvente para a mulher e família tanto quanto menos confiável para a equipe.	Concordo totalmente	Concordo totalmente	Concordo totalmente	Concordo	Concordo totalmente
Gostei muito da iniciativa e do trabalho criado acredito que vai contribuir muito para melhorar a assistência. Apenas acho importante trazer ou ao longo do conteúdo ou no final da cartilha as referências utilizadas para construir e o ano desse material afim de demostrar a população que a informação oferecida foi algo baseado nas evidências mais atuais.	Concordo totalmente	Concordo totalmente	Concordo totalmente	Concordo totalmente	Concordo totalmente

	Concordo totalmente	Concordo totalmente	Concordo totalmente	Concordo totalmente	Concordo totalmente
-Tentar deixar os textos mais curtos, com palavras maiores (para não fiar cansativa a leitura) e com linguajar mais leve.<br>-Se atentar para pequenos erros de português e concordância, que talvez seja interessante ser revisado por um especialista em línguas;<br>-Na página 11 há uma recomendação de que nenhum profissional coloque as mãos na vulva, isso precisa ser revisto, pois as diretrizes de assistência ao parto do MS tem a recomendação de "mãos sobre" que é uma escolha do profissional segurar a cabeça do bebê ou não, e se isso fizer parte de sua assistência, mesmo com recomendação e a paciente não souber ou entender direito a maneira como isso se dá, pode se sentir desrespeitada, sendo que não foi esse o objetivo;<br>-Colocar "Pique" entre aspas já que é uma linguagem informal;<br>-Quanto aos graus de laceração, será que se faz realmente necessário colocá-los? não seria mais eficaz explicar apenas que existem lacerações mais ou menos profundas, e que em algumas vezes precisará de sutura para preservação da anatomia e prevenção de hemorragia?! Penso que saber os graus de laceração cabe mais aos profissionais da assistÊncia ao parto do que ás mulheres;<br>-Em relação ao manejo da placenta, a diretriz nacional de assistência ao parto, orienta tanto o manejo ativo quanto o manejo fisiológico, e recomenda inclusive o manejo ativo para prevenção de HPP, é bom rever a palavra "ideal" para manejo fisiológico, pois nem sempre será o ideal.	Concordo parcialment e	Concordo totalmente	Concordo totalmente	Concordo parcialmente	Concordo parcialmente
Deve falar mais sobre o pré natal do homem	Concordo	Concordo	Concordo	Concordo	Concordo
Cartilha ficou extensa	Concordo	Concordo	Concordo	Concordo totalmente	Concordo
	Concordo	Concordo totalmente	Concordo totalmente	Concordo	Concordo

	Concordo	Concordo totalmente	Concordo totalmente	Concordo	Concordo
	Concordo totalmente	Concordo totalmente	Concordo totalmente	Concordo totalmente	Concordo totalmente
	Concordo	Concordo totalmente	Concordo	Concordo	Concordo
As sugestões foram enviadas pelo Whats App da autora.	Concordo	Concordo parcialment e	Concordo totalmente	Concordo parcialmente	Concordo parcialmente
	Concordo	Concordo	Concordo	Concordo	Concordo
	Concordo totalmente	Concordo totalmente	Concordo	Concordo	Concordo
Abordar sobre a ruptura das membranas, quando procurar o hospital, se é preciso que a bolsa "estoure" para o bebê nascer	Concordo totalmente	Concordo totalmente	Concordo totalmente	Concordo totalmente	Concordo totalmente

	Concordo	Concordo	Concordo	Concordo	Concordo
A página 09 está duplicada.<br>Parabéns pelo conteúdo riquíssimo!! Será um elo muito importante para discussões dos assuntos mais pertinentes à gestantes durante seu acompanhamento pré-natal !	Concordo totalmente	Concordo	Concordo totalmente	Concordo	Concordo
Não ha sugestões sobre os capítulos existentes, porém seria interessante a inclusão de capítulo falando sobre prematuridade que é algo que acontece com certa frequencia e as mulheres não estão preparadas	Concordo	Concordo	Concordo totalmente	Concordo totalmente	Concordo
	Concordo totalmente	Concordo totalmente	Concordo totalmente	Concordo totalmente	Concordo
Poderia finalizar falando da sexualidade pós-parto, quando iniciar, autocuidado, desejos e medos e ansiedades. E também poderia abordar com uma pequena contribuição sobre ansiedade e puerpério.	Concordo totalmente	Concordo totalmente	Concordo totalmente	Concordo totalmente	Concordo

3.6 A linguagem está adequada ao público alvo.	3.7 As idéias estão expressas concisamente.	3.8 O texto possibilita interação com orientações entre profissionais e público alvo.	3.9 O texto possibilita interação com o encademaneto lógico do processo de parturição.	3.10 O planejamento e a sequência das informações são consistentes, facilitando o entendimento pelo público alvo.	3.11 O material é de leitura agradével.	3.12 O material tem tamanho adequado, ou seja, não é extenso e nem cansativo.
Concordo totalmente	Concordo totalmente	Concordo totalmente	Concordo totalmente	Concordo totalmente	Concordo totalmente	Concordo totalmente
Concordo	Concordo	Concordo	Concordo	Concordo	Concordo	Concordo
Concordo totalmente	Concordo	Concordo totalmente	Concordo	Concordo totalmente	Concordo totalmente	Concordo
Concordo totalmente	Concordo	Concordo totalmente	Concordo	Concordo totalmente	Concordo totalmente	Concordo totalmente
Concordo parcialmente	Concordo	Concordo totalmente	Concordo totalmente	Concordo totalmente	Concordo totalmente	Concordo totalmente
Concordo totalmente	Concordo totalmente	Concordo totalmente	Concordo totalmente	Concordo totalmente	Concordo totalmente	Concordo totalmente

Concordo totalmente	Concordo totalmente	Concordo totalmente	Concordo totalmente	Concordo totalmente	Concordo totalmente	Concordo
Concordo parcialmente	Concordo	Concordo	Concordo totalmente	Concordo	Concordo	Concordo parcialmente
Concordo	Concordo	Concordo	Concordo	Concordo	Concordo	Concordo
Concordo	Concordo	Concordo	Concordo	Concordo	Concordo	Concordo parcialmente
Concordo	Concordo	Concordo	Concordo	Concordo	Concordo	Concordo

Concordo	Concordo totalmente	Concordo totalmente	Concordo totalmente	Concordo totalmente	Concordo totalmente	Concordo parcialmente
Concordo totalmente	Concordo totalmente	Concordo totalmente	Concordo totalmente	Concordo totalmente	Concordo totalmente	Concordo totalmente
Concordo totalmente	Concordo totalmente	Concordo totalmente	Concordo totalmente	Concordo totalmente	Concordo totalmente	Concordo totalmente
Concordo parcialmente	Discordo totalmente	Concordo totalmente	Concordo totalmente	Concordo parcialmente	Concordo parcialmente	Discordo totalmente
Concordo parcialmente	Concordo	Concordo	Concordo	Concordo	Concordo	Concordo
Concordo	Concordo totalmente	Concordo totalmente	Concordo totalmente	Concordo totalmente	Concordo totalmente	Concordo
Concordo totalmente	Concordo totalmente	Concordo totalmente	Concordo totalmente	Concordo totalmente	Concordo	Concordo

Concordo	Concordo	Concordo	Concordo	Concordo	Concordo	Concordo
Concordo	Concordo	Concordo totalmente	Concordo totalmente	Concordo	Concordo	Concordo
Concordo	Concordo	Concordo	Concordo	Concordo	Concordo	Concordo parcialmente
Concordo	Concordo parcialmente	Concordo	Concordo	Concordo	Concordo	Concordo
Concordo totalmente	Concordo totalmente	Concordo totalmente	Concordo totalmente	Concordo totalmente	Concordo totalmente	Concordo parcialmente

Acrescente sua sugestão quanto à linguagem da cartilha neste espaço (por favor especifique o capítulo):	4.1 As ilustrações são simples, apropriadas e de tracejado de fácil compreensão.	4.2 As ilustrações são familiares para os leitores.	4.3 Estão relacionadas com o texto (configuram o propósito desejado).	4.5 Estão integradas ao texto (bem localizadas).	4.6 São autoexplicativas.
	Concordo totalmente	Concordo totalmente	Concordo totalmente	Concordo totalmente	Concordo totalmente
	Concordo	Concordo	Concordo	Concordo	Concordo
	Concordo totalmente	Concordo	Concordo totalmente	Concordo	Concordo
Informações com facilidade de entendimento	Concordo totalmente	Concordo totalmente	Concordo	Concordo totalmente	Concordo totalmente
já fiz acima	Concordo	Concordo totalmente	Concordo	Concordo totalmente	Concordo totalmente
	Concordo totalmente	Concordo totalmente	Concordo totalmente	Concordo totalmente	Concordo totalmente

	Concordo totalmente	Concordo totalmente	Concordo totalmente	Concordo totalmente	Concordo totalmente
Quanto a linguagem neutra; a questão de colocar questões bíblicas e religiosas é algo que precisa ser revisto. Um material de saúde precisa ser imparcial quanto a isso e respeitar todas as opiniões religiosas.	Concordo totalmente	Concordo totalmente	Concordo totalmente	Concordo totalmente	Concordo totalmente
Achei boa a abordagem	Concordo	Concordo totalmente	Concordo totalmente	Concordo totalmente	Concordo totalmente
	Concordo	Concordo totalmente	Concordo	Concordo	Concordo
	Concordo totalmente	Concordo	Concordo	Concordo totalmente	Concordo

Algumas partes como a sobre a fase ativa de dilatação e do período expulsivo estão bem longas e com mais termos técnicos em relação as outras partes do texto, podendo diminuir o interesse pela leitura ou não permitir o esclarecimento completo sem ajuda de um profissional (Ex: em uma parte do texto é falado sobre avaliação da vitalidade fetal e materna, que não é uma linguagem habitual; ou quando é trazida informações sobre rotação do feto antes do desprendimento final, acredito que a informação seja bastante técnica, poderia sugerir trazer apenas que o feto pode não sair rapidamente e de uma só vez, podendo ocorrer saída da cabeça fetal e em uma próxima contração acontecer a saída do corpo)	Concordo totalmente	Concordo totalmente	Concordo totalmente	Concordo totalmente	Concordo totalmente
	Concordo totalmente	Concordo totalmente	Concordo totalmente	Concordo totalmente	Concordo totalmente
	Concordo totalmente	Concordo totalmente	Concordo totalmente	Concordo totalmente	Concordo totalmente
Material muito extenso, com assuntos repwtidos. Observações mais detalhadas foram enviadas pelo whatApp da pesquisadora	Concordo parcialmente	Concordo parcialmente	Concordo parcialmente	Concordo totalmente	Concordo parcialmente
	Concordo totalmente	Concordo	Concordo	Concordo	Concordo parcialmente
	Concordo	Concordo parcialmente	Concordo	Concordo	Concordo
	Concordo totalmente	Concordo totalmente	Concordo totalmente	Concordo totalmente	Concordo totalmente

	Concordo	Concordo	Concordo	Concordo	Concordo
	Concordo totalmente	Concordo totalmente	Concordo totalmente	Concordo totalmente	Concordo totalmente
	Concordo	Concordo	Concordo	Concordo	Concordo parcialmente
	Concordo totalmente	Concordo totalmente	Concordo totalmente	Concordo totalmente	Concordo
Uma cartilha bem extensa. As figuras também deixam um pouco cansativo.	Concordo parcialmente	Concordo	Concordo	Concordo	Concordo

Acrescente sua sugestão quanto às ilustrações da cartilha neste espaço (por favor especifique o capítulo):	5.1 O material promove o entendimento para se evitar a cirurgia cesariana desnecessária.	5.2 Propicia o máximo de informações para a redução de cesarianas desnecessárias e aumento das taxas de partos normais.	5.3 As instruções para a obtenção de um parto normal respeitoso são claras e compreensíveis.	5.4 O uso de sentido dúbido não ocorre no texto.	5.5 O conteúdo é escrito em estilo que tem a gestante/parturiente como centro.	Acrescente sua sugestão quanto ao layout da cartilha neste espaço (por favor especifique o capítulo):
	Concordo totalmente	Concordo totalmente	Concordo totalmente	Concordo totalmente	Concordo totalmente	
	Concordo	Concordo	Concordo	Concordo	Concordo	
	Concordo	Concordo	Concordo	Concordo totalmente	Concordo totalmente	
	Concordo totalmente	Concordo	Concordo	Concordo	Concordo totalmente	
só a figura da pagina 2 que parece não ornar com o contexto	Concordo totalmente	Concordo totalmente	Concordo totalmente	Concordo parcialmente	Concordo totalmente	Já fiz mais acima. Pelo estudo da mente humana se sabe que precisa ter o cuidado da palavra "não", para que o cérebro na sua rapidez faça realmente a leitura negativa.
Não sei se caberia nesse contexto de cartilha de acrescentar a fonte da onde foram retiradas as imagens.	Concordo totalmente	Concordo totalmente	Concordo totalmente	Concordo totalmente	Concordo totalmente	

	Concordo totalmente	Concordo totalmente	Concordo totalmente	Concordo totalmente	Concordo totalmente	
	Concordo totalmente	Concordo totalmente	Concordo parcialmente	Concordo parcialment e	Concordo	
Ter mais sobre a equipe de apoio	Concordo totalmente	Concordo totalmente	Concordo totalmente	Concordo	Concordo	Boa apresentação
	Concordo	Concordo	Concordo	Concordo	Concordo	
	Concordo totalmente	Concordo	Concordo	Concordo	Concordo	

	Concordo totalmente	Concordo totalmente	Concordo totalmente	Concordo totalmente	Concordo totalmente	Na página 20, possui um erro de digitação no quadro (41 semanas a 41 semanas e 6 dias)
	Concordo totalmente	Concordo totalmente	Concordo totalmente	Concordo totalmente	Concordo totalmente	
	Concordo parcialmente	Concordo parcialmente	Concordo	Concordo totalmente	Concordo totalmente	Na minha opinião a cartilha ajudará na orientação para evolução adequado de um parto normal com mínimo de, tervenções, mas não será suficiente para diminuir as taxas alarmantes de cesariana realizada no nosso estado. Seria importante informar sobre as iatrogenias causadas para a mãe e para o bebê, como problemas pulmonares, efeitos da imaturidade e as consequências tanto para o bebê e para a mãe que não entrou em contato com os hormônios do parto.
Precisa rever as figuras 10, 14, 21 e 29	Concordo totalmente	Concordo	Concordo parcialmente	Concordo totalmente	Concordo parcialmente	Sugestões enviadas pelo whats App
	Concordo parcialmente	Concordo parcialmente	Concordo	Concordo	Concordo	
	Concordo totalmente	Concordo totalmente	Concordo totalmente	Concordo	Concordo totalmente	
	Concordo totalmente	Concordo totalmente	Concordo totalmente	Concordo	Concordo totalmente	

	Concordo	Concordo	Concordo	Concordo	Concordo	
	Concordo totalmente	Concordo totalmente	Concordo	Concordo totalmente	Concordo totalmente	
	Concordo	Concordo	Concordo parcialmente	Concordo	Concordo	
	Concordo totalmente	Concordo totalmente	Concordo totalmente	Concordo	Concordo	
	Concordo	Concordo	Concordo	Concordo	Concordo	

6.1 O tamanho da fonte é adequada.	6.2 O estilo das letras é adequado.	6.3 O espaçamento das letras é adequado.	6.4 O comprimento das linhas é adequado.	6.5 O espaçamento entre linhas é adequado.	6.6 Existe uso adequado do espaço em branco para reduzir a aparência de texto abarrotado.	6.7 O espaçamento entre parágrafos é adequado.	6.8 O formato do material é adequado.	Acrescente sua sugestão quanto ao layout da cartilha neste espaço (por favor especifique o capítulo):	7.1 Está inserida na cultura local.
Concordo totalmente	Concordo totalmente	Concordo totalmente	Concordo totalmente	Concordo totalmente	Concordo totalmente	Concordo totalmente	Concordo totalmente		Concordo totalmente
Concordo	Concordo	Concordo	Concordo	Concordo	Concordo	Concordo	Concordo		Concordo
Concordo	Concordo	Concordo	Concordo	Concordo	Concordo	Concordo	Concordo		Concordo
Concordo	Concordo	Concordo totalmente	Concordo totalmente	Concordo	Concordo	Concordo totalmente	Concordo totalmente		Concordo totalmente
Concordo totalmente	Concordo totalmente	Concordo totalmente	Concordo totalmente	Concordo totalmente	Concordo totalmente	Concordo totalmente	Concordo totalmente	amei tudo do layout	Concordo totalmente
Concordo totalmente	Concordo totalmente	Concordo totalmente	Concordo totalmente	Concordo totalmente	Concordo totalmente	Concordo totalmente	Concordo totalmente		Concordo

Concordo totalmente	Concordo totalmente	Concordo totalmente	Concordo totalmente	Concordo totalmente	Concordo totalmente	Concordo totalmente	Concordo totalmente		Concordo totalmente
Concordo parcialmente	Concordo parcialmente	Concordo parcialmente	Concordo	Concordo parcialmente	Concordo parcialmente	Concordo parcialmente	Concordo	Como já colocado, acredito que o tamanho da fonte em algumas páginas está muito pequeno, o que dificulta e torna a leitura cansativa, principalmente para o público mais leigo.	Concordo
Concordo	Concordo	Concordo	Concordo	Concordo	Concordo	Concordo	Concordo	Boa e fácil leitura	Concordo
Concordo totalmente	Concordo totalmente	Concordo totalmente	Concordo totalmente	Concordo totalmente	Concordo totalmente	Concordo	Concordo		Concordo
Concordo	Concordo	Concordo	Concordo	Concordo	Concordo	Concordo	Concordo		Concordo

Concordo totalmente	Concordo totalmente	Concordo totalmente	Concordo totalmente	Concordo totalmente	Concordo	Concordo	Concordo totalmente		Concordo totalmente
Concordo totalmente	Concordo totalmente	Concordo totalmente	Concordo totalmente	Concordo totalmente	Concordo totalmente	Concordo totalmente	Concordo totalmente		Concordo totalmente
Concordo totalmente	Concordo totalmente	Concordo totalmente	Concordo totalmente	Concordo totalmente	Concordo totalmente	Concordo totalmente	Concordo totalmente		Concordo parcialmente
Discordo totalmente	Concordo totalmente	Concordo totalmente	Concordo totalmente	Concordo parcialmente	Concordo	Concordo parcialmente	Concordo totalmente	Sugiro retirar as citações bíblicas	Concordo parcialmente
Concordo	Concordo	Concordo	Concordo	Concordo	Concordo	Concordo	Concordo		Concordo
Concordo	Concordo	Concordo	Concordo	Concordo	Concordo	Concordo	Concordo		Concordo
Concordo	Concordo	Concordo totalmente	Concordo totalmente	Concordo totalmente	Concordo	Concordo	Concordo		Concordo

Concordo	Concordo	Concordo	Concordo	Concordo	Concordo	Concordo	Concordo		Concordo
Concordo parcialmente	Concordo totalmente	Concordo	Concordo	Concordo	Concordo	Concordo	Concordo		Concordo
Concordo parcialmente	Concordo	Concordo parcialmente	Concordo parcialmente	Concordo parcialmente	Concordo parcialmente	Concordo parcialmente	Concordo	Texto mais espaçado dividido em etapas com ilustrações explicativas, algumas paginas tem muito texto o que pode tornar desinteressante para algumas pessoas	Concordo parcialmente
Concordo totalmente	Concordo totalmente	Concordo totalmente	Concordo totalmente	Concordo totalmente	Concordo	Concordo	Concordo		Concordo
Concordo parcialmente	Concordo parcialmente	Concordo parcialmente	Concordo parcialmente	Concordo parcialmente	Concordo parcialmente	Concordo parcialmente	Concordo		Concordo

7.2 Está inserida na cultura atual.	7.3 O material habilita o público alvo a realizar as ações desejadas.	7.4 O material ajuda a prevenir possíveis problemas no parto.	7.5 O material permite obter o máximo benefício possível.	Acrescente sua sugestão neste espaço (por favor especifique o capítulo):	8.1 O que você gostou na cartilha?
Concordo totalmente	Concordo totalmente	Concordo totalmente	Concordo totalmente		Informativa, auto explicativa , instrumento que reside na possibilidade de escolhas consciente pelo tipo de parto e assistência.
Concordo	Concordo	Concordo	Concordo		Tudo! Bem sucinto e explicativo
Concordo	Concordo	Concordo	Concordo		Clareza e didática
Concordo totalmente	Concordo totalmente	Concordo	Concordo		Conteúdo literário
Concordo totalmente	Concordo totalmente	Concordo totalmente	Concordo totalmente		Achei o trabalho fantástico, de um valor inestimável, alguns pontos estratégicos a meu ver para ajustar, mas de maneira geral é um ótimo instrumento.
Concordo	Concordo totalmente	Concordo totalmente	Concordo totalmente	Talvez por estamos inseridas no MS, um estado que tem uma parte da população indígena principalmente no interior do estado. poderia haver/ trazer um capítulo ou parte que traga algo relacionado a essa população.	Eu gostei muito!!! Um trabalho bem feito, trazendo evidências mais atualizadas, numa linguagem clara e acessível a mulheres gestantes que através da leitura podem se munir de informações para garantia dos seus direitos durante o gestar e parir no nosso país de tantas desigualdades.<br>Ótimo trabalho!!!

Concordo totalmente	Concordo totalmente	Concordo totalmente	Concordo totalmente		Ótima em todos os sentidos. Parabéns pela iniciativa.
Concordo	Concordo totalmente	Concordo	Concordo		A cartilha é de grande valia para que mais pessoas gestantes tenham acesso a um conteúdo que nem sempre é passado em consultas tradicionais. Incentiva o autocuidado e pra que a mulher seja protagonista de seu parto, que é a base da humanização. Tem ilustrações interessantes e que chama a atenção, além de possibilitar novos debates e diálogos sobre essa temática.<br>Parabéns pela iniciativa!
Concordo	Concordo	Concordo	Concordo	Boa apresentação	Precisamos de material simples e de fácil leitura com informações verdadeiras
Concordo	Concordo	Concordo parcialmente	Concordo		A explicação sobre os pródomos que é o que mais leva a mulher a buscar atendimento nas maternidades. Falsas indicações de cesariana e sobre os métodos não farmacológico.
Concordo	Concordo	Concordo	Concordo		Sim

Concordo totalmente	Concordo totalmente	Concordo totalmente	Concordo totalmente		Particularmente, gostei muito da produção desse material. Percebe-se, ao logo de toda a cartilha, o cuidado com que a pesquisadora teve em buscar e apresentar as melhores e mais recentes evidências científicas para composição de um material educativo com linguagem simples, acessível, objetiva e de qualidade. Acredito que a cartilha mostra-se uma ferramenta efetiva e importante para educação em saúde obstétrica para usuárias e também para capacitar e atualizar profissionais de saúde e áreas afins.
Concordo totalmente	Concordo totalmente	Concordo totalmente	Concordo totalmente		apresenta clara e objetiva uma linguagem de fácil acesso
Concordo parcialmente	Concordo totalmente	Concordo parcialmente	Concordo totalmente	Na minha opinião o tema não está inserido nem cultura local e atual, pois, nosso estado e país o número de cesariana e muito elevado, existe sim uma cultura de cesariana, no estado 63% das crianças nascem de cesariana.<br>A cartilha ajuda diminuir as intervenções desnecessárias, mas não evita a ocorrências de intercorrências que não pode ser previsto nem pelo profissional e pela mãe, como a distócia de ombro e hemorragia pós-parto.<br>Sugiro que conste na cartilha sobre os efeitos nocivos da manobra de Kristeller e a iatrogenia causado pela cesariana para a mãe e para o bebê.	Gostei da orientação detalhada da sequência da evolução trabalho de parto, pois, sabemos que muitas mulheres pensam que chegou nas 40 semanas e iniciou as primeiras contrações de treinamento pensa que já está em trabalho de parto e já começa contar o tempo de trabalho de parto. Seria importante constar também na cartilha que o trabalho de parto se divide em três fases: pródromos, fase de latência (1-5cm de dilatação), fase ativa (6-10cm fase ativa) e período expulsivo.
Concordo parcialmente	Concordo	Concordo	Concordo		Do conteúdo.
Concordo	Concordo	Concordo	Concordo		Conceito e as figuras
Concordo	Concordo	Concordo	Concordo totalmente		Sim. Muito bem elaborada e educativa para incentivo ao parto normal.
Concordo	Concordo totalmente	Concordo parcialmente	Concordo		A forma prática e de fácil entendimento como o processo do parto foi abordado.<br>As figuras demonstrando o funcionamento das contrações em relação ao colo uterino.<br>Abordagem dos mitos e verdades sobre as cesarianas.

Concordo	Concordo	Concordo totalmente	Concordo totalmente		Explicação em linguagem apropriada para quem não sabe os termos técnicos.
Concordo totalmente	Concordo	Concordo	Concordo totalmente		Conteúdo riquíssimo que embasará uma boa conduta entre usuárias e profissionais de saúde com estímulo à prática mais humanizadas !
Concordo	Concordo	Concordo parcialmente	Concordo parcialmente		É um material bem completo com linguagem simples e de fácil compreensão
Concordo totalmente	Concordo	Concordo	Concordo totalmente		A cartilha está escrita de forma coesa e concisa. As ilustrações ao longo do texto complementam as informações, fazendo com que a gestante entenda melhor como seu corpo funciona durante o trabalho de parto. Além de desencorajar cesárias sem real necessidade, a cartilha expõe o parto normal como um processo fisiológico que faz parte e complementa o desfecho da gestação. Este é um instrumento que a gestante pode consumir com calma durante a gestação e pode complementar o pré-Natal, havendo uma troca de informações bilateral entre profissional e paciente.
Concordo	Concordo	Concordo	Concordo		Explicativo e informativo. Irá promover o poder de decisão e escolha de mulheres e suas famílias.

Nome	IDADADE GESTACIONAL (trimestre)	Sexo	Estado Civil	PARIDADE	1.a A capa chamou sua atenção	1.b A sequência do conteúdo está adequada	1.c A estrutura da cartilha educativa está adequada
G01	3	Feminino	Casada	PRIMIGESTA	Adequado	Adequado	Adequado
G02	2	Feminino	Solteira	PRIMIGESTA	Adequado	Parcialmente Adequado	Adequado
G03	3	Feminino	Casada	SECUNDIGESTA	Adequado	Adequado	Parcialmente Adequado
G04	3	Feminino	Casada	MULTIGESTA	Adequado	Adequado	Adequado
G05	3	Feminino	Casada	PRIMIGESTA	Adequado	Adequado	Adequado
G06	3	Feminino	Casada	SECUNDIGESTA	Adequado	Adequado	Adequado
G07	3	Feminino	Casada	PRIMIGESTA	Parcialmente Adequado	Adequado	Adequado
G08	3	Feminino	Casada	PRIMIGESTA	Adequado	Adequado	Adequado
G09	2	Feminino	Solteira	PRIMIGESTA	Adequado	Adequado	Adequado
G10	3	Feminino	Solteira	PRIMIGESTA	Adequado	Adequado	Adequado
G11	3	Feminino	Casada	PRIMIGESTA	Adequado	Adequado	Adequado
G12	2	Feminino	Casada	MULTIGESTA	Adequado	Adequado	Adequado
G13	3	Feminino	Casada	PRIMIGESTA	Adequado	Adequado	Adequado
G14	2	Feminino	Casada	PRIMIGESTA	Adequado	Adequado	Adequado
G15	2	Feminino	Casada	SECUNDIGESTA	Adequado	Adequado	Adequado
G16	2	Feminino	Casada	SECUNDIGESTA	Adequado	Adequado	Adequado
G17	2	Feminino	Casada	PRIMIGESTA	Parcialmente Adequado	Adequado	Adequado
G18	2	Feminino	Casada	PRIMIGESTA	Parcialmente Adequado	Adequado	Adequado
G19	2	Feminino	Casada	PRIMIGESTA	Adequado	Adequado	Adequado
G20	3	Feminino	Casada	SECUNDIGESTA	Adequado	Adequado	Adequado
G21	3	Feminino	Solteira	PRIMIGESTA	Adequado	Adequado	Adequado
G22	2	Feminino	Solteira	SECUNDIGESTA	Adequado	Adequado	Adequado

1.d O conteúdo destaca os pontos principais	2.a As frases são fáceis de entender	2.b O conteúdo escrito é claro	2.c Usa vocabulário com palavras comuns no texto	2.d O aprendizado é facilitado por tópicos	2.e O texto é interessante	3.a O propósito da ilustração referente ao texto está claro	3.b As ilustrações são simples
Adequado	Adequado	Adequado	Adequado	Adequado	Adequado	Adequado	Adequado
Adequado	Adequado	Adequado	Adequado	Adequado	Adequado	Adequado	Parcialmente Adequado
Adequado	Adequado	Adequado	Adequado	Adequado	Adequado	Adequado	Parcialmente Adequado
Adequado	Adequado	Parcialmente Adequado	Parcialmente Adequado	Adequado	Adequado	Adequado	Adequado
Adequado	Adequado	Adequado	Adequado	Adequado	Adequado	Adequado	Adequado
Adequado	Adequado	Adequado	Adequado	Adequado	Adequado	Adequado	Adequado
Adequado	Adequado	Adequado	Adequado	Parcialmente Adequado	Adequado	Adequado	Adequado
Adequado	Adequado	Adequado	Adequado	Adequado	Adequado	Adequado	Adequado
Adequado	Adequado	Adequado	Adequado	Adequado	Adequado	Adequado	Parcialmente Adequado
Adequado	Adequado	Adequado	Parcialmente Adequado	Adequado	Adequado	Adequado	Adequado
Adequado	Adequado	Adequado	Adequado	Adequado	Adequado	Adequado	Adequado
Adequado	Adequado	Parcialmente Adequado	Adequado	Adequado	Adequado	Parcialmente Adequado	Parcialmente Adequado
Parcialmente Adequado	Adequado	Adequado	Adequado	Adequado	Adequado	Parcialmente Adequado	Parcialmente Adequado
Adequado	Adequado	Adequado	Adequado	Adequado	Adequado	Adequado	Adequado
Adequado	Adequado	Adequado	Adequado	Adequado	Adequado	Adequado	Adequado
Adequado	Adequado	Adequado	Adequado	Adequado	Adequado	Adequado	Adequado
Adequado	Adequado	Adequado	Adequado	Adequado	Adequado	Adequado	Adequado
Adequado	Adequado	Adequado	Adequado	Adequado	Parcialmente Adequado	Parcialmente Adequado	Adequado
Adequado	Adequado	Parcialmente Adequado	Adequado	Parcialmente Adequado	Adequado	Adequado	Adequado
Adequado	Adequado	Adequado	Adequado	Adequado	Adequado	Adequado	Adequado
Adequado	Adequado	Adequado	Adequado	Adequado	Parcialmente Adequado	Adequado	Adequado
Adequado	Adequado	Adequado	Adequado	Adequado	Adequado	Adequado	Adequado

3.c As ilustrações servem para complementar o texto?	3.d As figuras/ilustrações são relevantes	4.a Característica do layout	4.b Tamanho e tipo de letra	4.c São utilizados subtítulos	4.d As páginas ou seções parecem organizadas	5.a Em sua opinião, qualquer gestante que ler essa cartilha vai entender?
Adequado	Adequado	Adequado	Adequado	Adequado	Adequado	Adequado
Adequado	Adequado	Adequado	Adequado	Adequado	Adequado	Adequado
Adequado	Adequado	Adequado	Parcialmente Adequado	Adequado	Adequado	Parcialmente Adequado
Adequado	Adequado	Adequado	Adequado	Adequado	Adequado	Adequado
Adequado	Adequado	Adequado	Adequado	Adequado	Adequado	Adequado
Adequado	Adequado	Adequado	Adequado	Adequado	Adequado	Adequado
Adequado	Parcialmente Adequado	Adequado	Adequado	Adequado	Adequado	Adequado
Adequado	Adequado	Adequado	Adequado	Adequado	Adequado	Adequado
Adequado	Parcialmente Adequado	Adequado	Adequado	Adequado	Parcialmente Adequado	Adequado
Adequado	Adequado	Adequado	Parcialmente Adequado	Adequado	Adequado	Parcialmente Adequado
Adequado	Adequado	Adequado	Adequado	Adequado	Adequado	Adequado
Adequado	Adequado	Adequado	Adequado	Adequado	Adequado	Adequado
Adequado	Adequado	Adequado	Adequado	Adequado	Adequado	Parcialmente Adequado
Adequado	Parcialmente Adequado	Adequado	Adequado	Adequado	Adequado	Adequado
Parcialmente Adequado	Adequado	Adequado	Adequado	Parcialmente Adequado	Adequado	Adequado
Adequado	Adequado	Adequado	Adequado	Adequado	Adequado	Adequado
Adequado	Parcialmente Adequado	Adequado	Adequado	Adequado	Adequado	Adequado
Adequado	Adequado	Adequado	Parcialmente Adequado	Adequado	Parcialmente Adequado	Adequado
Adequado	Adequado	Parcialmente Adequado	Adequado	Adequado	Adequado	Adequado
Adequado	Adequado	Adequado	Adequado	Adequado	Adequado	Adequado
Parcialmente Adequado	Adequado	Adequado	Adequado	Adequado	Adequado	Adequado
Adequado	Adequado	Adequado	Adequado	Adequado	Adequado	Adequado

5.b No material educativo são abordados os assuntos necessários para que as gestantes sintam-se preparadas para o trabalho de parto e parto.	5.c Você se sentiu motivado a ler a cartilha até o final	6.a O material é culturalmente adequado à sua lógica, linguagem e experiência	6.b Apresenta imagens e exemplos adequados culturalmente	Sugestões/Comentários
Adequado	Adequado	Adequado	Adequado	
Adequado	Parcialmente Adequado	Adequado	Adequado	
Adequado	Adequado	Adequado	Adequado	
Adequado	Adequado	Adequado	Adequado	
Adequado	Adequado	Adequado	Adequado	
Adequado	Adequado	Adequado	Adequado	Cartilha tirou muitos mitos que tinha na minha cabeça. Com certeza terei meu segundo parto mais informada e tranquila.
Adequado	Adequado	Adequado	Adequado	
Adequado	Adequado	Adequado	Adequado	
Adequado	Adequado	Adequado	Adequado	
Adequado	Adequado	Adequado	Adequado	
Adequado	Adequado	Adequado	Adequado	
Adequado	Adequado	Adequado	Adequado	
Parcialmente Adequado	Adequado	Adequado	Adequado	
Adequado	Adequado	Adequado	Adequado	
Adequado	Adequado	Adequado	Adequado	
Adequado	Adequado	Adequado	Adequado	
Adequado	Adequado	Adequado	Adequado	
Adequado	Adequado	Adequado	Adequado	
Adequado	Adequado	Adequado	Adequado	
Adequado	Adequado	Parcialmente Adequado	Adequado	
Adequado	Adequado	Adequado	Adequado	
Adequado	Adequado	Adequado	Adequado	
