# Supplementary material for: Educational booklet on labor and delivery: validity study
Source: Rev Bras Enferm. 2024 Dec 13;77(5):e20240138. doi: 10.1590/0034-7167-2024-0138 (PMC11654228; doi:10.1590/0034-7167-2024-0138)
Supplement: Translation

# Translation: Cartilha educativa sobre trabalho de parto e parto: estudo de validade

**DOI:** 10.1590/0034-7167-2024-0138pt

**Authors:** Michelle Tatiane Carvalho Gonçalves, Ana Roberta Vilarouca da Silva, Mara Cristina Ribeiro Furlan, Bruna Moretti Luchesi, Tatiana Carvalho Reis Martins

**Affiliations:** I Universidade Federal de Mato Grosso do Sul. Três Lagoas, Mato Grosso do Sul, Brasil; II Universidade Federal do Piauí. Picos, Piauí, Brasil; III Universidade Federal de Mato Grosso do Sul. Campo Grande, Mato Grosso do Sul, Brasil

**Keywords:** Educação em Saúde, Estudos de Validação, Cuidado Pré-Natal, Saúde da Mulher, Parto Humanizado

## Abstract

**Objetivos:**

construir e identificar evidências de validade de cartilha educativa sobre trabalho de parto e parto para gestantes.

**Métodos:**

estudo metodológico, com construção e validação de uma cartilha baseada no referencial de Echer. A validação de conteúdo e aparência adotou o Índice de Validade de Conteúdo e o alfa de Cronbach, tendo juízes selecionados pelo critério de Fering. Depois, realizou-se a validação clínica com a população-alvo.

**Resultados:**

a cartilha, construída com base em evidências de uma revisão integrativa, validada com juízes e público-alvo, obteve Índice de Validade de Conteúdo global de 0,919 e 0,913 respectivamente. Após validação clínica com 22 gestantes, foi apresentada em 28 tópicos e 48 páginas, com ilustrações produzidas por designer gráfico.

**Conclusões:**

a presente tecnologia educativa foi considerada válida pelos juízes especialistas e pelo público-alvo, portanto é considerada uma ferramenta relevante na promoção da saúde das gestantes.

## INTRODUÇÃO

A gestação caracteriza-se como um período de grandes mudanças físicas e psicológicas na vida de uma mulher, resultando em maior vulnerabilidade e sensibilidade emocional^(1)^. É durante o pré-natal que a mulher e sua família têm a oportunidade de sanar dúvidas, minimizando aflições e construindo um caminho mais seguro no decorrer da gestação^(2)^. Nesse sentido, uma assistência adequada ao longo da gestação é imprescindível para um desfecho positivo e manutenção do bem estar materno e fetal, promovendo saúde e prevenindo agravos^(3)^.

No Brasil, a assistência às gestantes é, em sua maioria, realizada por profissionais médicos. Esse modelo resulta em uma assistência altamente medicalizada e intervencionista. Evidências dessa situação são dadas por pesquisas atuais mostrando baixa adesão às boas práticas na assistência ao trabalho de parto por parte desses profissionais^(4,5)^. Assim, a consulta pré-natal, que deveria ser um momento para educação perinatal, não vem sendo aproveitada em sua máxima capacidade. No tocante a essas consultas, muitas fragilidades têm sido reveladas, tais como falta de vínculo entre as gestantes e profissionais assistentes, consultas rápidas e genéricas ocasionadas pelas grandes demandas das unidades de saúde e ausência de outras categorias profissionais capacitadas para atender esse público específico^(6)^.

No pré-natal, gestantes são submetidas a exames mensais desnecessários e hipermedicalização. Nas rotinas hospitalares, mulheres são impedidas de se alimentar, de deambular, de estar na presença de seu acompanhante, de acessar métodos não farmacológicos de alívio da dor e analgesia. Além disso, são expostas a uma alta taxa de intervenções obstétricas, como acesso venoso, ocitocina, amniotomia, litotomia, episiotomia e cesárea sem real indicação. Alguns estudos têm sugerido insatisfação por parte das mulheres em relação à assistência recebida no decurso do trabalho de parto e parto - relatos incluindo queixas de abuso, desrespeito e maus-tratos são comuns nos serviços de saúde^(7,8)^.

Considerando a necessidade de ofertar uma assistência qualificada e humanizada, é preciso estabelecer ações que ajudem as mulheres e suas famílias a experimentarem de forma plena e positiva a gestação, parto e puerpério. Especificamente, o acesso à informação de qualidade durante o pré natal pode mostrar se uma ferramenta eficaz, contribuindo para desfechos positivos de parto e nascimento, além de diminuir sentimentos como medo, ansiedade e sofrimento^(2)^.

Estabelecer estratégias educativas na assistência às gestantes, como a criação de uma cartilha, poderá minimizar as dificuldades de entendimento. Isso viabiliza uma comunicação mais clara entre as mulheres e profissionais, com garantia de uma assistência mais acolhedora capaz de suprir as necessidades ligadas à gestação, trazendo confiança, segurança, autonomia e protagonismo à mulher^(2)^.

No contexto da saúde da mulher em seu ciclo gravídico e puerperal, constata-se a importância de elaborar e validar um material educativo impresso (cartilha) de fácil compreensão e acessível, com temas relevantes sobre as fases do trabalho de parto e parto^(9)^. A validação é um processo importante para garantir a produção de tecnologias educacionais de alta qualidade, com linguagem acessível ao público-alvo e alcance dos objetivos propostos^(10)^.

A cartilha, como instrumento de informação mediante orientações claras, pode subsidiar a criação e desenvolvimento de ações educativas durante todo o ciclo gravídico-puerperal, mostrando se uma poderosa ferramenta de reforço às orientações verbalizadas pelos profissionais de saúde^(10)^. Quanto a isso, bons resultados tem sido observados, como o aumento da satisfação, conhecimento e adesão ao processo de tratamento e autocuidado após o uso do material impresso, trazendo impactos positivos na relação profissional/paciente^(11)^. Além do mais, o uso de material educativo apresenta-se como uma tecnologia emancipatória, pois, ao ofertar informações, contribui no empoderamento dos indivíduos e favorece o processo de comunicação e orientação entre a equipe de saúde, pacientes e familiares^(9)^.

Considerando o exposto, este estudo partiu da hipótese de que essa tecnologia educativa contribuirá para consultas de pré-natal qualificadas, propiciando desfechos positivos de parto e fazendo com que o conhecimento adquirido pela mulher torne-a protagonista nesse processo. Haja vista a necessidade de mudanças do cenário obstétrico referente à assistência ao trabalho de parto e parto, esperam-se efeitos positivos, pois as mulheres serão agentes de transformação dentro das unidades hospitalares com base na percepção e conhecimento do processo de parturição, aguçando nos profissionais assistentes a busca por saberes pautados nas boas práticas de assistência ao parto.

## OBJETIVOS

Construir e identificar evidências de validade de cartilha educativa sobre trabalho de parto e parto para gestantes.

## MÉTODOS

### Aspectos éticos

O projeto está em consonância com os termos da Resolução nº 466/2012, do Conselho Nacional de Saúde. O Comitê de Ética em Pesquisa com Seres Humanos da Universidade Federal de Mato Grosso do Sul aprovou esta pesquisa. Todos os indivíduos envolvidos no estudo assinaram o Termo de Consentimento Livre e Esclarecido (TCLE), por meio on-line e por escrito.

### Desenho e período do estudo

Trata-se de estudo com abordagem metodológica, para validar uma cartilha educativa. A pesquisa foi realizada entre setembro de 2022 e maio de 2023, em quatro etapas: 1) levantamento bibliográfico; 2) elaboração das ilustrações, do layout, do design e dos textos; 3) validação aparente e de conteúdo do material educativo por juízes especialistas no assunto; e 4) validação do material por representantes do público-alvo^(12)^. O estudo foi estruturado de acordo com o *Revised Standards for Quality Improvement Reporting Excellence* (SQUIRE 2.0)^(13)^.

### Etapas de desenvolvimento

Na primeira etapa, foi realizado um levantamento bibliográfico para embasar a construção da cartilha a fim de proporcionar segurança e respaldo no uso da tecnologia educativa. Para definir os temas abordados na cartilha, utilizaram-se as recomendações da OMS; Associação Brasileira de Obstetrizes e Enfermeiros Obstetras (ABENFO); Diretrizes Nacionais de Parto Normal^(14)^; Comissão Nacional de Incorporação de Tecnologias (CONITEC) do Sistema Único de Saúde (SUS), Diretrizes de Atenção às Gestantes: operação cesariana^(15)^; *American Association of Birth Centers* (AABC); *Royal College of Obstetricans and Gynaecologists* (RCOG); e *National Institute for Health and Clinical Excellence* (NICE).

Na segunda etapa, foi elaborado o roteiro do conteúdo a ser abordado, com informações importantes sobre o trabalho de parto e parto, além de sugestões de ilustrações que representem bem o conteúdo e as características do público-alvo. Houve cuidado em relação à adequação da linguagem técnica para uma linguagem mais popular, visando facilitar a compreensão pelas gestantes. A diagramação da cartilha e estruturação textual ocorreram com base nas recomendações referentes à escrita e formatação de texto de tecnologias educativas^(16)^. As ilustrações foram retiradas do banco gratuito de ilustrações e imagens do aplicativo CANVA e do perfil de Instagram da fisioterapeuta Laura Delega (@assoalhopelvico) mediante autorização.

A terceira etapa constituiu-se do processo de validação da aparência e conteúdo do material educativo por juízes especialistas no assunto. O cálculo amostral para determinação da quantidade de juízes foi obtido por meio da fórmula *n = Za^2^ × P(1-P) / e^2^*. Os valores estipulados foram “Za” (nível de confiança) = 95%, “P” (proporção de concordância dos juízes) = 85%, bem como “e” (diferença aceita do que se espera) = 15%^(17)^. O cálculo final foi estabelecido em *n = (1,962 × 0,85 × 0,15) / 0,152*, o que resultou em 22.

Foram selecionados os participantes que atuavam no ensino, assistência e pesquisa envolvendo a enfermagem obstétrica de acordo com adaptações do modelo Fehring^(18)^. Os critérios para seleção dos juízes especialistas no assunto foram: ter especialização ou residência em obstetrícia (3 pontos), ter no mínimo um ano de experiência assistencial em obstetrícia (2 pontos), ter experiência docente na área de obstetrícia (2 pontos), ter publicação na área de obstetrícia (2 pontos), ter mestrado (3 pontos) e ter participado como ouvinte de capacitações na área de obstetrícia (2 pontos)^(18)^. Foi realizada uma consulta ao Currículo Lattes (disponível on-line na Plataforma Lattes) para verificar a adequação do especialista aos critérios estabelecidos no estudo. Foram selecionados aqueles que obtiveram, no mínimo, sete pontos. Os primeiros especialistas participantes do estudo faziam parte de um grupo de WhatsApp de enfermeiros obstetras e ginecologistas do estado do Mato Grosso do Sul. Todos receberam o convite. Aqueles que demonstraram interesse em participar da pesquisa foram contatados em particular, via WhatsApp ou e-mail, e eles foram indicando outros potenciais juízes.

A carta-convite para participar da pesquisa foi enviada por e-mail (pessoal ou institucional) ou WhatsApp. Aos juízes que aceitaram, foi enviada (por e-mail ou WhatsApp) uma versão da cartilha, o instrumento de validação e uma cópia do TCLE. O instrumento de validação foi elaborado com o auxílio do aplicativo *Google Forms*.

O instrumento de coleta de dados dos juízes foi dividido em duas partes. A primeira continha questões referentes à caracterização do perfil, compreendendo os dados socioeconômicos e dados profissionais relacionados à atuação, capacitação, produção científica e docência envolvendo a obstetrícia. A segunda parte do instrumento referia-se à validação da cartilha. O instrumento foi elaborado com base em outras pesquisas^(2,19)^, com 52 itens distribuídos em sete aspectos avaliativos que correspondem ao conteúdo, linguagem, relevância, ilustrações, layout, motivação e cultura. Em cada item, a avaliação é realizada por meio de uma escala do tipo Likert com quatro opções disponíveis: 1) discordo totalmente, 2) concordo parcialmente, 3) concordo e 4) concordo totalmente^(2,19)^. Ao final do instrumento, solicitou-se aos juízes que realizassem comentários gerais sobre a cartilha. Essa coleta de dados ocorreu entre setembro e dezembro de 2022.

Na quarta etapa, procedeu-se à validação clínica com as gestantes. O número de convidados para essa avaliação foi estabelecido pela mesma fórmula determinante da amostra de juízes (n = 22). Os critérios de inclusão foram: ser maior de 18 anos; ser gestante atendida nas Unidades de Saúde da Família da cidade de Três Lagoas, Mato Grosso do Sul; e saber ler e escrever. Foram excluídas as gestantes que tinham alguma deficiência visual ou cognitiva que impossibilitasse a leitura da cartilha. A abordagem foi realizada nas unidades durante os momentos de maior fluxo de gestantes, as quais ali estavam para passar por consulta médica ou de enfermagem. O convite era realizado pessoalmente pelo pesquisador. Àquelas que aceitavam participar da pesquisa, ali mesmo já lhes era explicado sobre conceitos éticos e termo de consentimento. Além disso, elas recebiam uma pasta plástica contendo uma caneta e o material impresso (TCLE, instrumento de validação e cartilha).

A coleta de dados com o público-alvo deu-se entre abril e maio de 2023. Foi entregue às gestantes uma cópia impressa da cartilha, o questionário e o TCLE. O questionário contemplou questões de caracterização e o instrumento adaptado do *Suitability Assessment of Materials* (SAM). O SAM é composto por 22 questões acerca das seguintes áreas: 1) conteúdo, 2) exigência de alfabetização, 3) ilustrações, 4) layout e apresentação, 5) estimulação/motivação para o aprendizado e 6) adequação cultural. Os itens são avaliados por uma escala do tipo Likert (0 = inadequado, 1 = parcialmente adequado e 2 = adequado)^(20,21)^.

### Análise de dados

Os dados dos juízes especialistas foram baixados no Excel e exportados para o *Statistical Package for the Social Sciences* (SPSS), versão 26.0. Já os dados do público-alvo foram digitados no Excel e exportados para o SPSS. Realizou-se a estatística descritiva por meio da frequência relativa, média e desvio-padrão. Para análise dos dados de validação do material educativo, foi realizado o cálculo do Índice de Validade de Conteúdo (IVC) para cada item da escala; depois, foram calculados o IVC médio entre os itens e o IVC global. O IVC maior ou igual a 80% foi considerado parâmetro de validade^(22)^. O alfa de Cronbach foi utilizado para medir a consistência interna do instrumento. Um coeficiente alfa de Cronbach entre 0,6 e 0,7 indica um nível aceitável de confiabilidade, enquanto coeficientes de 0,8 ou acima apontam um nível muito bom de confiabilidade^(23)^.

## RESULTADOS

### Produção da cartilha educativa

Na primeira etapa, foi realizada a busca na literatura especializada mediante um levantamento de dados nos materiais elaborados pelo Ministério da Saúde, OMS, colégios nacionais e internacionais de ginecologia e obstetrícia e associações nacionais e internacionais de enfermagem obstétrica e ginecologia. Iniciou-se o levantamento de dados pelas diretrizes do Ministério da Saúde, pois essa instituição conduz no Brasil as orientações inerentes à prática assistencial à saúde da população. Realizou-se também busca em banco de dados. A primeira busca encontrou 1.060 artigos. Após a seleção, procedeu-se à leitura dos respectivos resumos, sendo a amostra final composta de 32 artigos. Os critérios de elegibilidade foram: boas práticas na assistência ao trabalho de parto e parto; humanização do parto; e boas práticas na assistência ao recém-nascido. A busca foi feita por meio do portal de periódicos da Coordenação de Aperfeiçoamento de Pessoal de Nível Superior (CAPES), nas bases de dados *Medical Literature Analyses and Retrieval System Online* (MEDLINE), Literatura Latino-Americana em Ciências da Saúde (LILACS), *National Library of Medicine* - *National Institutes of Health* (PubMed), *Scopus e Web of Science* (WoS). Utilizou-se o método de busca avançada, categorizando título, resumo e assunto. Procedeu-se à leitura e, após, iniciou-se a elaboração textual, na qual se buscou oferecer um conteúdo detalhado, mas sem perder o objetivo de uma linguagem acessível ao público-alvo.

Na segunda etapa, foram realizadas a elaboração e a organização dos tópicos e assuntos do material educativo, bem como a escolha do título. A cartilha foi intitulada “CADA PARTO É UMA HISTÓRIA: orientações e dicas sobre as fases do trabalho de parto”. Foi composta por capa e 47 páginas com tamanho padrão de formatação de 21 cm de altura por 15 cm de largura (Figura 1). Optou-se por apresentá-la em 23 tópicos: o parto é seu, seja protagonista da sua história; a chegada do bebê; o que saber sobre pródromos; tampão mucoso; plano de parto; fases de abertura-latente; fases de abertura ativa; curiosidade: como se proteger dos estímulos do neocórtex; partolândia - fase de transição; fase do expulsivo e bolsa das águas; não precisa cortar para nascer - episiotomia; e se rasgar? Laceração; dequitação - nascimento da placenta; dor no parto; confiança no parto; métodos não farmacológicos no alívio da dor; falsas indicações de cesariana; indicações reais de cesariana; o primeiro contato - contato pele a pele; como o bebê fica logo após o nascimento; uma boa hora de ouro e as primeiras horas após o parto. Ao final da cartilha, para garantir maior interatividade, foram elaborados um modelo de plano de parto, um guia para deixar o pós-parto mais tranquilo, uma lista com o que levar para a maternidade e duas listas com indicações de filmes, documentários e canais no YouTube relacionados a parto e maternidade.

**Figura 1:** Capa e páginas da cartilha educativa produzida e validada neste estudo, Três Lagoas, Mato Grosso do Sul, Brasil, 2023

A terceira etapa foi constituída pela seleção das ilustrações e adequação do vocabulário, finalizando com a diagramação. Como ilustrações, foram escolhidas imagens dinâmicas e atrativas e fotos reais de partos que transparecessem a verdade de como deve ser uma assistência ao parto com boas práticas. Houve ainda o cuidado de selecionar imagens com boa qualidade e coloridas. Elas foram retiradas do banco gratuito de ilustrações e imagens do aplicativo CANVA, do perfil de Instagram da fisioterapeuta Laura Della Negra (@assoalhopélvico) mediante autorização e do acervo pessoal de atendimentos da autora, também mediante autorização para uso de imagem. Quanto ao vocabulário, buscou-se empregar palavras de fácil entendimento e coerentes com a proposta textual a ser transmitida ao público-alvo. A diagramação da cartilha e estruturação textual ocorreram com base nas recomendações referentes à escrita e formatação de texto de tecnologias educativas^(16)^.

### Validação por especialistas

Nessa etapa, 23 juízes especialistas aceitaram participar da pesquisa de avaliação do material educativo. A maioria dos profissionais eram do sexo feminino (90,9%), casados ou viviam com companheiro (60,9%) e tinham média de idade de 37,5 anos (DP = 9,19). Quanto à unidade federativa de atuação, houve predomínio do Mato Grosso do Sul (78,3%), seguido da Paraíba (8,7%), Minas Gerais (4,3%), Rio Grande do Norte (4,3%) e São Paulo (4,3%). Sobre a formação profissional, todos haviam cursado especialização em Ginecologia e Obstetrícia, 95,7% tinham capacitação em Ginecologia e Obstetrícia, sendo que 77,3% realizaram a última capacitação nos anos de 2021/2022. No tocante à titulação, 30,4% tinham mestrado; e 8,7%, doutorado.

No processo de validação da tecnologia educativa, todos os itens atingiram os critérios mínimos de aprovação (IVC ≥ 80%), sendo que o IVC foi superior a 0,91 em seis das sete categorias avaliadas. Com um IVC global de 0,91, o material educativo foi considerado validado (Tabela 1).

**Tabela 1:** Avaliação do Índice de Validade de Conteúdo e avaliação da consistência interna das respostas dos profissionais enfermeiros (N = 23), Três Lagoas, Mato Grosso do Sul, Brasil, 2023

	DT	CP	C	CT	IVC	Alfa de Cronbach
	n	n	n	n		
1. Exatidão científica					0,942	0,879
1.1 Os conteúdos abordados estão de acordo com o conhecimento atual?	0	1	6	16	0,957	0,846
1.2 As orientações apresentadas são as necessárias e foram abordadas corretamente?	0	1	11	11	0,957	0,872
1.3 Os termos técnicos estão adequadamente definidos?	0	2	6	15	0,913	0,752
2. Conteúdo					0,930	0,857
2.1 Os objetivos das informações são evidentes (levar informação clara às gestantes)?	0	2	5	16	0,913	0,822
2.2 As informações são satisfatórias quanto ao comportamento desejado?	0	1	10	12	0,957	0,814
2.3 Não existe informações desnecessárias.	1	2	11	9	0,870	0,785
2.4 Existe revisão dos pontos mais importantes.	0	2	9	12	0,913	0,835
2.5 As infrações são atualizadas.	0	0	5	18	1,000	0,903
3. Apresentação literária					0,928	0,912
3.1 A linguagem é neutra (sem adjetivos comparativos, sem ser promocional e sem apelos inverídicos).	0	1	12	10	0,957	0,906
3.2 A linguagem é explicativa.	0	1	7	15	0,957	0,901
3.3 O material promove e encoraja a adesão ao parto normal.	0	0	7	16	1,000	0,918
3.4 O vocabulário é composto em sua maioria por palavras comuns.	0	2	10	11	0,913	0,907
3.5 O vocabulário empregado é composto por palavras simples.	0	2	13	8	0,913	0,897
3.6 A linguagem está adequada ao público-alvo.	0	4	10	9	0,826	0,900
3.7 As ideias estão expressas concisamente.	1	1	12	9	0,913	0,902
3.8 O texto possibilita interação com orientações entre profissionais e público-alvo.	0	0	9	14	1,000	0,905
3.9 O texto possibilita interação com o encadeamento lógico do processo de parturição.	0	0	10	13	1,000	0,915
3.10 O planejamento e a sequência das informações são consistentes, facilitando o entendimento pelo público-alvo.	0	1	10	12	0,957	0,892
3.11 O material é de leitura agradável.	0	1	11	11	0,957	0,896
3.12 O material tem tamanho adequado, ou seja, não é extenso nem cansativo.	1	5	11	6	0,739	0,910
4. Ilustrações					0,930	0,889
4.1 As ilustrações são simples, apropriadas e de fácil compreensão.	0	2	7	14	0,913	0,882
4.2 As ilustrações são familiares para os leitores.	0	2	7	14	0,913	0,843
4.3 Estão relacionadas com o texto (configuram o propósito desejado).	0	1	10	12	0,957	0,850
4.5 Estão integradas ao texto (bem localizadas).	0	0	8	15	1,000	0,888
4.6 São autoexplicativas.	0	3	8	12	0,870	0,849
5. Material suficientemente específico e compreensivo					0,913	0,733
5.1 O material promove o entendimento para se evitar a cirurgia cesariana desnecessária.	0	2	6	15	0,913	0,680
5.2 Propicia o máximo de informações para a redução de cesarianas desnecessárias e aumento das taxas de partos normais.	0	2	9	12	0,913	0,618
5.3 As instruções para a obtenção de um parto normal respeitoso são claras e compreensíveis.	0	3	10	10	0,870	0,628
5.4 O uso de sentido dúbio não ocorre no texto.	0	2	12	9	0,913	0,806
5.5 O conteúdo é escrito em estilo que tem a gestante/parturiente como centro.	0	1	10	12	0,957	0,678
6. Legibilidade e características da impressão					0,875	0,952
6.1 O tamanho da fonte é adequado.	1	4	9	9	0,783	0,949
6.2 O estilo das letras é adequado.	0	2	10	11	0,913	0,946
6.3 O espaçamento das letras é adequado.	0	3	8	12	0,870	0,938
6.4 O comprimento das linhas é adequado.	0	2	9	12	0,913	0,941
6.5 O espaçamento entre linhas é adequado.	0	4	9	10	0,826	0,938
6.6 Existe uso adequado do espaço em branco para reduzir a aparência de texto abarrotado.	0	3	13	7	0,870	0,938
6.7 O espaçamento entre parágrafos é adequado.	0	4	12	7	0,826	0,942
6.8 O formato do material é adequado.	0	0	14	9	1,000	0,954
7. Qualidade da informação					0,913	0,846
7.1 Está inserida na cultura local.	0	3	14	6	0,870	0,769
7.2 Está inserida na cultura atual.	0	2	13	8	0,913	0,808
7.3 O material habilita o público-alvo a realizar as ações desejadas.	0	0	13	10	1,000	0,858
7.4 O material ajuda a prevenir possíveis problemas no parto.	0	4	12	7	0,826	0,804
7.5 O material permite obter o máximo benefício possível.	0	1	11	11	0,957	0,834
IVC global					0,919	

*DT - discordo totalmente; CP - concordo parcialmente; C - concordo; CP - concordo parcialmente; IVC - Índice de Validade de Conteúdo.*

Quanto à consistência interna, o material educativo obteve em seis categorias avaliadas valores indicando um nível muito bom de confiabilidade. Apenas a Categoria 5, “Material suficientemente específico e compreensivo”, mostrou coeficiente alfa de 0,73, que representa um nível aceitável de confiabilidade (Tabela 1).

Os juízes deram 27 sugestões, das quais 21 foram aceitas: 15 referentes ao conteúdo, para melhoria na clareza do texto; 1 relativa à linguagem, para troca de termos técnicos por termos mais populares; 4 relacionadas às ilustrações, para a inclusão e mudança de ilustração mais conivente com o texto; e 1 ligada ao layout, para diminuir a extensão do texto e dar maior espaçamento entre parágrafos, deixando-os mais limpos visualmente.

As sugestões e comentários dos juízes foram de grande valia, enriquecendo e aprimorando o material educativo para que este alcançasse o objetivo proposto. Pontos principais foram destacados: a escolha do tema, classificado como muito pertinente e relevante; o uso de recentes evidências científicas; a riqueza de detalhes nas informações; e a linguagem de fácil compreensão.

Na avaliação da consistência interna, ainda que o quesito “Material suficientemente específico e compreensivo”, da Categoria 5, tenha tido coeficiente alfa de 0,73 (nível aceitável de confiabilidade), os juízes sugeriram alterações para o aperfeiçoamento do material educativo, as quais foram aceitas.

Notadamente sobre o conteúdo da cartilha, os juízes deram sugestões relevantes: incluir o apoio e presença do acompanhante em todo o processo de informação durante a gestação; substituir a frase “nenhum profissional coloque as mãos na vulva” [durante o parto], visto que o certo, como consta nas Diretrizes de Assistência ao Parto do Ministério da Saúde, é “mãos sobre”, sendo, portanto, uma escolha do profissional segurar a cabeça do bebê ou não; substituir as informações a respeito dos graus de laceração por uma explicação mais simples e de fácil entendimento; quanto ao manejo da placenta, a diretriz nacional de assistência ao parto orienta tanto o manejo ativo quanto o manejo fisiológico e recomenda inclusive o manejo ativo para prevenção de hemorragia pós-parto, substituindo, logo, a palavra “ideal” por “manejo fisiológico”; incluir o tema “ruptura de membranas (bolsa das águas)”; e incluir informações sobre o puerpério.

No que tange à escrita, os juízes julgaram: realizar a troca de alguns termos técnicos por termos mais simples para facilitar a compreensão do público-alvo - por exemplo, substituir “avaliação da vitalidade fetal e materna” e “rotação do feto antes do desprendimento final”, respectivamente, por “ avaliar os batimentos do coraçãozinho do bebê e o bem-estar da mãe” e “o bebê poderá não sair rapidamente e de uma só vez, podendo ocorrer saída da cabeça e, em uma próxima contração, a saída do corpo”; substituir a palavra “não” em algumas frases, tendo em vista a dificuldade do cérebro em assimilar tal palavra; além da realização de revisão textual.

No que se refere às ilustrações, os juízes comentaram sobre a necessidade de acrescentar as referências das imagens. No tocante à adequação cultural, apenas um juiz comentou que a proposta da cartilha não está inserida na cultura local e atual, pois o estado do Mato grosso do Sul e o Brasil têm altas taxas de cesariana, estabelecendo, portanto, uma cultura “cesarista”. É necessário enfatizar que, nos comentários e/ou sugestões, foram apontados problemas de ortografia e concordância; dessa forma, o material passou por revisão textual por profissional habilitado.

### Validação clínica

Participaram dessa etapa de validação 22 gestantes atendidas em cinco eSFs de Três Lagoas. A maioria das participantes eram casadas (77,3%), com idade entre 25 e 30 anos e tinha cursado o ensino superior (53%). Em relação à paridade, 14 (63,6%) eram primigestas, 6 (27,3%) eram secundigestas, e 2 (9,1%) eram multigestas. Quanto à idade gestacional, 10 (45,5%) estavam no segundo trimestre, e 12 (54,5%) estavam no terceiro. Sobre os dados obstétricos, observou-se que a minoria das gestantes (43,0%) realizou sete ou mais consultas de pré-natal, fato justificado pela idade gestacional das mulheres em acompanhamento de pré-natal.

Das seis categorias avaliadas pelas gestantes, cinco obtiveram um IVC superior a 0,91, e somente a Categoria 3, “Ilustrações”, apresentou valor menor (0,84). O IVC total da cartilha foi de 0,91, alcançando o valor necessário para ser validado (Tabela 2).

**Tabela 2:** Avaliação do Índice de Validade de Conteúdo pelas gestantes (N = 22), Três Lagoas, Mato Grosso do Sul, Brasil, 2023

	Inadequado	Parcialmente adequado	Adequado	IVC
	n	n	n	
1 - Conteúdo				0,932
1.a A capa chamou sua atenção.	0	3	19	0,864
1.b A sequência do conteúdo está adequada.	0	1	21	0,955
1.c A estrutura da cartilha educativa está adequada.	0	1	21	0,955
1.d O conteúdo destaca os pontos principais.	0	1	21	0,955
2 - Estilo de escrita				0,918
2.a As frases são fáceis de entender.	0	0	22	1,000
2.b O conteúdo escrito é claro.	0	3	19	0,864
2.c Usa vocabulário com palavras comuns no texto.	0	2	20	0,909
2.d O aprendizado é facilitado por tópicos.	0	2	20	0,909
2.e O texto é interessante.	0	2	20	0,909
3 - Ilustrações				0,841
3.a O propósito da ilustração referente ao texto está claro.	0	3	19	0,864
3.b As ilustrações são simples.	0	5	17	0,773
3.c As ilustrações servem para complementar o texto.	0	2	20	0,909
3.d As figuras/ilustrações são relevantes.	0	4	18	0,818
4 - Layout e apresentação				0,920
4.a Característica do layout	0	1	21	0,955
4.b Tamanho e tipo de letra	0	3	19	0,864
4.c São utilizados subtítulos.	0	1	21	0,955
4.d As páginas ou seções parecem organizadas.	0	2	20	0,909
5 - Estimulação/Motivação do aprendizado				0,924
5.a Em sua opinião, qualquer gestante que ler essa cartilha vai entender?	0	3	19	0,864
5.b No material educativo são abordados os assuntos necessários para que as gestantes se sintam preparadas para o trabalho de parto e parto.	0	1	21	0,955
5.c Você se sentiu motivado a ler a cartilha até o final.	0	1	21	0,955
6 - Adequação cultural				0,977
6.a O material é culturalmente adequado à sua lógica, linguagem e experiência.	0	1	21	0,955
6.b Apresenta imagens e exemplos adequados culturalmente.	0	0	22	1,000
IVC global				0,913

*IVC - Índice de Validade de Conteúdo.*

Nos itens avaliados, percebeu-se que todas as gestantes conseguiram ter bom entendimento sobre a temática da cartilha e seu objetivo principal. Quase todas (95,5%) classificaram a cartilha como material necessário para que se sintam preparadas para o trabalho de parto e parto. Sobre o texto, todas entenderam as frases contidas na cartilha. Ainda, observou-se que a maioria das gestantes (95,5%) disse ter tido interesse em realizar a leitura da cartilha até o final. Além disso, 95,5% afirmaram que o conteúdo destaca os principais pontos. Apenas uma gestante realizou sugestão/comentário: “*A cartilha tirou muitos mitos que tinha na minha cabeça. Com certeza, terei meu segundo parto mais informada e tranquila*”.

Após a validação clínica, a versão final da cartilha foi composta de 48 páginas, sendo 44 destinadas ao conteúdo e 4 páginas pós-textuais.

## DISCUSSÃO

No presente estudo, um material educativo foi produzido e validado no formato de cartilha. O uso dessas tecnologias validadas concede maior confiabilidade, facilitando a comunicação no processo ensino-aprendizagem em saúde^(24)^. Corriqueiramente os materiais educativos são utilizados na assistência à saúde abarcando um vasto repertório de assuntos^(25)^. Além disso, encontram-se diferentes formatos dessas tecnologias, tais como manuais para execução de uma cirurgia segura; jogos educativos sobre depressão na adolescência; álbum seriado sobre sífilis congênita; cartilha educativa para o cuidado domiciliar ao recém-nascido prematuro; folder educativo para coleta de escarro da tuberculose pulmonar; vídeo educativo sobre cuidados ao recém-nascido; dentre outros^(2-26)^.

Essas tecnologias de educação em saúde desempenham um papel importante na promoção à saúde. Elas têm capacidade de mudar realidades individuais e coletivas e levar mais consciência nas tomadas de decisões, diminuindo possíveis riscos à saúde e promovendo desfechos mais favoráveis^(27)^.

O levantamento bibliográfico foi um passo essencial na construção desse material educativo. A revisão de literatura buscou as mais recentes evidências sobre a temática, tendo como norte as diretrizes apontadas pelo Ministérios da Saúde sobre assistência ao parto no Brasil. Por fim, as ideias apresentadas foram convergentes com as necessidades do público-alvo, alcançando o objetivo proposto^(27)^.

Na validação pelos juízes, a cartilha mostrou-se confiável com um bom ICV, demonstrando estar adequada ao público-alvo. A maioria das sugestões e recomendações foram acatadas com objetivo de melhorar a linguagem e compreensão das informações apresentadas. Já as sugestões não acatadas dizem respeito àquelas já citadas anteriormente, e uma apenas não estava de acordo com a proposta da cartilha. Assim, as principais modificações realizadas segundo as sugestões dos juízes referem-se ao conteúdo, envolvendo acréscimo de alguns assuntos ausentes e substituição de certos termos técnicos por outros mais compreensíveis pelo público-alvo. As respostas das gestantes foram muito positivas, destacando-se que consideraram a cartilha interessante e informativa.

O grande diferencial dessa cartilha em comparação com outras disponíveis é a riqueza de detalhes e aprofundamento no tema, com dicas práticas de “o que fazer e o que irá sentir” em cada fase do trabalho de parto, tornando esse material singular. Como estratégia para chamar a atenção das gestantes, foram utilizados tópicos como “Curiosidade”, também presente em outros materiais educativos^(10,28)^.

No Brasil, a assistência ao parto é hospitalar em sua totalidade, num modelo medicalocêntrico. Como consequência dessa abordagem, tal assistência torna-se altamente intervencionista e medicalizada. Isso é confirmado por pesquisas atuais mostrando baixa adesão às boas práticas na assistência ao trabalho de parto e ao parto por parte dos profissionais condutores da assistência^(4,5)^. Nas rotinas hospitalares, mulheres são privadas de se alimentar, de deambular, de estar na presença de seu acompanhante, de acessar métodos não farmacológicos de alívio da dor e analgesia, sendo submetidas a uma alta taxa de intervenções obstétricas, como acesso venoso, ocitocina, amniotomia, litotomia, episiotomia e cesárea sem real indicação. Alguns estudos têm sugerido insatisfação por parte das mulheres no tocante à assistência recebida durante o trabalho de parto e parto, com relatos queixas de abuso, desrespeito e maus-tratos sendo comuns nos serviços de saúde^(7,8)^.

Dessa forma, a cartilha educativa “CADA PARTO É UMA HISTÓRIA: orientações e dicas sobre as fases do trabalho de parto” é uma tecnologia educativa validada, pois contém linguagem, ilustrações e layout adequados, simples e atraentes para os leitores. Espera-se que a cartilha seja utilizada durante o pré-natal pelas gestantes e contribua para a melhoria da qualidade das consultas de pré natal, favorecendo desfechos positivos de parto e fazendo com que o conhecimento adquirido pela mulher torne-a protagonista nesse processo.

O uso desse material educativo apresenta-se ainda como uma tecnologia emancipatória, pois, ao ofertar informações, contribui no empoderamento dos indivíduos, além de favorecer o processo de comunicação e orientação entre a equipe de saúde, pacientes e familiares^(9)^.

### Limitações do estudo

Como limitação, destaca-se a dificuldade de obter uma amostra representativa de juízes de todos os estados do Brasil, havendo um maior número de participantes da Região Centro-Oeste.

### Contribuições para a área da saúde

Durante as consultas de pré-natal, o enfermeiro poderá utilizar também essa tecnologia educativa, a cartilha, para levar informação às gestantes a respeito dos conteúdos abordados. Além disso, ele pode usá-la para identificar riscos e vulnerabilidades dessa população, além de prestar-lhe apoio e esclarecimento, tendo a educação e a promoção à saúde como pilares na condução desse cuidado.

## CONCLUSÕES

Os juízes especialistas e o público-alvo consideraram relevantes o conteúdo e a aparência da tecnologia educativa, com o IVC total superior a 0,90 evidenciando valores satisfatórios. Ressalta-se que a metodologia aplicada foi capaz de auxiliar na elaboração da cartilha educativa de forma didática, dinâmica, compreensível, atrativa e dialógica, contribuindo para o conhecimento das mulheres gestantes e possivelmente também dos profissionais que exercem o cuidado a esse público.

Os resultados da validação por juízes especialistas e público-alvo evidenciam a adequação do conteúdo à realidade local, de modo que o material pode ajudar a preencher lacunas na assistência às gestantes. Destaca-se que esse material educativo tem potencial para colaborar com a redução das más práticas na assistência ao parto e diminuição da violência obstétrica.

## Data Availability

https://doi.org/10.48331/scielodata.HSJMFS
